# The Patterning Cascade Model and Human Mandibular Premolar Variation

**DOI:** 10.1002/ajpa.70178

**Published:** 2025-12-09

**Authors:** Molly Militello, Dori E. Kenessey, Christopher M. Stojanowski, Kathleen S. Paul

**Affiliations:** ^1^ Department of Anthropology University of Arkansas Fayetteville Arkansas USA; ^2^ School of Veterinary Medicine University of Wisconsin‐Madison Madison Wisconsin USA; ^3^ Department of Anthropology University of Nevada Reno Nevada USA; ^4^ Center for Bioarchaeological Research, School of Human Evolution and Social Change Arizona State University Tempe Arizona USA

**Keywords:** dental morphology, development, patterning cascade model, premolars

## Abstract

**Objectives:**

The patterning cascade model (PCM) provides a predictive framework for examining crown variation and posits that tooth size and enamel knot spacing, along with surrounding inhibitory fields, strongly influence crown morphology. The aim of this study is to determine if mandibular premolar variation aligns with predictions derived from the PCM.

**Materials and Methods:**

Using three‐dimensional (3D) scans and two‐dimensional (2D) images of dental casts representing a contemporary human sample (*n* = 63), we collected crown area, cusp area, and intercusp distance measurements, including 3D distances to account for differential cusp height. After controlling for size variation, relative measurements were subjected to analyses of variance, *t*‐tests, dichotomized generalized linear model regressions, and linear regressions to examine their relationship with lingual cusp number.

**Results:**

Relative intercusp distance between the mesiolingual cusp and its distal neighbor differs significantly between two and three lingual cusped premolars; those with two lingual cusps exhibit greater distance, on average. Two lingual cusped P_4_s exhibit greater 2D distance between the buccal and mesiolingual cusps. Models involving the relative area of the mesiolingual cusp and its distal neighbor were significant, with three lingual cusped P_4_s exhibiting smaller cusps.

**Discussion:**

These findings partially align with PCM‐derived expectations. The mesiolingual cusp is involved in all significant findings, which suggests its precursory enamel knot placement and inhibitory zoning have the greatest influence on mandibular premolar morphology. Generally, 2D and 3D analyses yield similar results, but the strength of the relationship is greater for 3D measurements in P_3_s, which are characterized by marked cusp height disparities.

## 1 Introduction

The patterning cascade model (PCM) posits that enamel knot spacing and tooth germ size influence final crown morphology (Jernvall and Jung 2000). This evolutionary‐developmental model originated through the study of the postcanine teeth of Lake Ladoga seals (*Phoca hispida ladogensis*), which consist of three to five cusps with significant height disparity aligned in a single row (Jernvall 2000). Subsequent studies of hominoid molars have provided mixed support for the model (Jernvall and Jung 2000; Hlusko et al. 2004; Hunter et al. 2010; Skinner and Gunz 2010; Moormann et al. 2013; Morita et al. 2016; Paul et al. 2017; Clark and Guatelli‐Steinberg 2018; Ortiz et al. 2018). However, unlike seal teeth, hominoid molars have a bulkier, quadrate, three‐dimensional form with primary cusps of fairly similar height.

Human mandibular premolars have been relatively overlooked in dental developmental studies. Yet as a tooth class, they represent an ideal PCM test case, as they vary in overall cusp number and exhibit considerable cusp height differences, similar to seal teeth (Jernvall 2000; Kocsis et al. 2002; Khraisat et al. 2013; Krenn et al. 2019). Here, we review concepts outlined in the PCM and examine the degree to which mandibular premolar variation conforms to predictions derived from the model using a sample of stone casts of human dentitions.

### 1.1 Overview of Odontogenesis

Odontogenesis—tooth development—consists of five stages: lamina, bud, cap, bell, and crown (Ten Cate 1994). This process is initiated by the formation of the primary dental lamina as epithelial thickenings curve along embryonic jaws (Ruch 1995; Jussila et al. 2014). Epithelial cells in the dental lamina proliferate, forming thickenings of oral epithelium called dental placodes. Each placode marks the site where a future tooth will develop (Ruch 1995; Thesleff 2006; Jussila et al. 2014). The bud stage occurs when the thickened oral epithelium invaginates into the neighboring ectomesenchyme layer, forming a tooth bud (Ruch 1995; Thesleff 2006).

Cap stage follows, during which (a) epithelial cells differentiate into the enamel organ, (b) inner mesenchyme differentiates into the dental papilla, and (c) outer mesenchyme differentiates into the dental follicle (Ruch 1995; Chiego 2013; Lesot et al. 2014). The enamel organ contains the inner enamel epithelium (IEE) and the outer enamel epithelium (OEE), which are joined at the cervical loop, the growing edge of the enamel organ (Ten Cate 1994; Thesleff and Tummers 2008; Chiego 2013). In cap stage, the primary enamel knot—the developmental precursor to the first forming tooth cusp—is initiated in the IEE (Butler 1956; Vaahtokari et al. 1996; Jernvall and Jung 2000; Jernvall and Thesleff 2000; Matalova et al. 2005). Enamel knots are regions of non‐dividing epithelial signaling centers that direct crown morphogenesis and cusp formation (Jernvall et al. 1994; Thesleff et al. 2001).

The bell stage follows the cap stage (Ruch 1995; Thesleff and Tummers 2008). In multicuspid teeth, this stage involves the formation of secondary enamel knots, which mark the sites of later developing cusps (Jernvall et al. 1994, 2000; Thesleff and Nieminen 1996; Jernvall 2000; Jernvall and Thesleff 2012). The IEE grows and folds around enamel knots defining the shape of each future cusp. In the late bell stage (or crown stage), the IEE differentiates into ameloblasts, enamel‐forming cells, and the dental papilla differentiates into odontoblasts, dentin‐forming cells (Ruch 1995; Thesleff and Tummers 2008; Jernvall and Thesleff 2012). Dentinogenesis and amelogenesis commence, with odontoblasts and ameloblasts originating at the enamel‐dentin junction (EDJ). Odontoblasts deposit dentin moving pulpward and ameloblasts deposit enamel, moving away from the pulp toward what will mark the outer enamel surface of the tooth crown (Ruch 1995; Thesleff and Tummers 2008).

#### 1.1.1 Enamel Knot Formation and Signaling

Four main signaling families play a role in enamel knot formation and ultimate cusp development: bone morphogenetic proteins (BMPs), fibroblast growth factor (FGF), wingless‐related integration site (WNT), and sonic hedgehog (SHH) (Jernvall et al. 1998; Kettunen and Thesleff 1998; Jernvall and Thesleff 2000; Thesleff 2006). By secreting related activator and inhibitor molecules, enamel knots impact multiple aspects of crown development (Salazar‐Ciudad and Jernvall 2002).

Following apoptosis of the primary enamel knot, activator molecules stimulate secondary enamel knot formation in multicuspid teeth (Jernvall et al. 1994, 2000; Thesleff and Nieminen 1996; Jernvall 2000; Jernvall and Thesleff 2012). Inhibitor molecules, meanwhile, prevent a new enamel knot from forming within the area surrounding an earlier‐forming enamel knot (Jernvall 2000; Jernvall and Thesleff 2000, 2012; Thesleff et al. 2001). The size of an inhibitory zone is determined by the nature of activator and inhibitor diffusion in the forming tooth germ (Salazar‐Ciudad and Jernvall 2002; Guatelli‐Steinberg et al. 2013). This activation–inhibition signaling activity continues in a *developmental cascade* (see Section 1.2 below), as subsequent secondary enamel knots arise (Jernvall 2000; Jernvall and Jung 2000; Guatelli‐Steinberg et al. 2013). Throughout, enamel knot signaling activity stimulates division of nearby epithelial and mesenchymal cells (Jernvall et al. 1994; Kettunen et al. 1998; Jernvall and Thesleff 2000; Thesleff et al. 2001), which contributes to IEE folding and determination of the crown's internal “blueprint” (Ten Cate 1994; Jernvall and Thesleff 2000; Thesleff et al. 2001).

As such, sequential enamel knot positioning and repeated signaling activity influence tooth form, including cusp configuration and intercusp distances (ICDs). Even small changes in the developmental timing and regulation of cusp formation can have a cumulative effect on crown morphology (Jernvall 2000; Jernvall and Jung 2000; Salazar‐Ciudad and Jernvall 2002), a phenomenon outlined in the PCM.

### 1.2 The Patterning Cascade Model

The PCM draws upon this understanding of enamel knot signaling and outlines the development of tooth morphology as a product of interactions between genes and select growth parameters, including tooth germ size, duration of crown formation, and initiation timing for individual cusps (Salazar‐Ciudad and Jernvall 2002; Hunter et al. 2010; Skinner and Gunz 2010). Importantly, the PCM has yielded a predictive framework for exploring crown variation in multicuspid teeth across various taxa (Jernvall 2000; Jernvall et al. 2000; Jernvall and Jung 2000). Jernvall's foundational study of seal molars demonstrated that variation in enamel knot spacing (approximated from cusp height) tracks variation in cusp number (Jernvall 2000). He found that variably present cusps on seal molars are typically shorter and smaller than those that are consistently present (Jernvall 2000). These late‐forming accessory cups are more likely to occur on blunt molars with relatively small height differences among early‐forming cusps, a configuration resulting from narrower inhibition zones surrounding enamel knots, as well as timely initiation of subsequent cusps (Jernvall 2000; Jernvall and Jung 2000).

However, the PCM acknowledges that gene signaling and iterative cusp development proceed within the spatial and temporal constraints of overall crown formation (Jernvall 2000; Hunter et al. 2010). A larger tooth germ has more area, and thus more cells with which an accessory enamel knot can develop (Jernvall 2000; Jernvall and Jung 2000). A larger tooth germ also requires a longer period of IEE growth, favoring the formation of more enamel knots and associated accessory cusps (Jernvall 2000; Jernvall and Jung 2000; Kondo and Townsend 2006; Harris 2007; Salazar‐Ciudad and Jernvall 2010). As such, researchers who have applied PCM‐derived predictions to primate dentitions have accounted for an interaction between crown size and cusp spacing as proxies for the time and space available for morphogenesis and enamel knot spacing, respectively (Hunter et al. 2010; Guatelli‐Steinberg et al. 2013). To do so, ICD measures have been used as proxies for enamel knot spacing and then statistically converted to relative intercusp distances (RICDs) through a calculation that accounts for crown size (e.g., Hunter et al. 2010; Moormann et al. 2013; Paul et al. 2017).

Over the last two decades, PCM‐derived predictions have been evaluated across a range of mammalian teeth with distinct crown morphologies and cusp arrangements (Jernvall 2000; Salazar‐Ciudad and Jernvall 2002). Variation in mice, vole, and seal teeth has supported predictions derived from the model (Keränen et al. 1998; Jernvall 2000; Salazar‐Ciudad and Jernvall 2002, 2010; Kavanagh et al. 2007). Numerous human studies have also yielded findings consistent with concepts outlined in the PCM. In a 2003 study of human mono‐ and dizygotic twins, Townsend and colleagues found that ICDs are highly variable and display marked levels of fluctuating asymmetry, indicating that the development of tooth morphology is affected by epigenetic/developmental events rather than solely additive genetic factors (Townsend et al. 2003). Similar results were yielded by more recent studies comparing cusp configuration and crown morphology in stressed versus non‐stressed groups (Riga et al. 2013) and nutritionally supplemented versus non‐supplemented groups (Blankenship‐Sefczek et al. 2024).

Hunter et al. (2010) also found that significant antimeric differences in Carabelli's trait expression in human maxillary first molars (M^1^s) are related to within‐individual antimeric differences in RICDs. These findings suggest that small developmental changes can have major effects on crown morphology independent of genotype (Hunter et al. 2010). Quantitative genetic studies provide similar indications—that non‐directional antimeric differences are influenced by environmental phenomena (Paul et al. 2023). Fluctuating asymmetry is often attributed to non‐genetic factors that may, in the case of crown morphology, include differential antimeric molecular signaling activity during crown development (Paul et al. 2023).

Collectively, though, direct tests of PCM‐derived predictions in primate dentitions have yielded mixed results (Hunter et al. 2010; Skinner and Gunz 2010; Guatelli‐Steinberg et al. 2013; Moormann et al. 2013; Morita et al. 2016; Paul et al. 2017; Clark and Guatelli‐Steinberg 2018; Kenessey et al. 2024). This suggests some complication in the application of PCM‐derived predictions to hominoid molars, although the cause is unclear. It may be due to aspects of their general crown morphology. Hominoid molars are characterized as bunodont, with rounded cusps organized across a relatively broad occlusal surface (Delezene and Ungar 2018). While developmental parameters are easily conceptualized along a single dimension in seal molars with linearly arranged cusps, the way in which zones of inhibition interact along multiple dimensions on quadrate molars may be more complex. Alternatively, discrepant findings may relate to the way in which spatial relationships among cusps are quantified. Some studies of hominoid molars focus solely on straight‐line, two‐dimensional (2D) distances between occlusal cusp tips (e.g., Hunter et al. 2010; Moormann et al. 2013; Kenessey et al. 2024), while others consider crown configuration in three dimensions (3D) at the enamel‐dentin junction (e.g., Skinner and Gunz 2010; Ortiz et al. 2018).

### 1.3 Mandibular Premolar Anatomy

**FIGURE 1:**
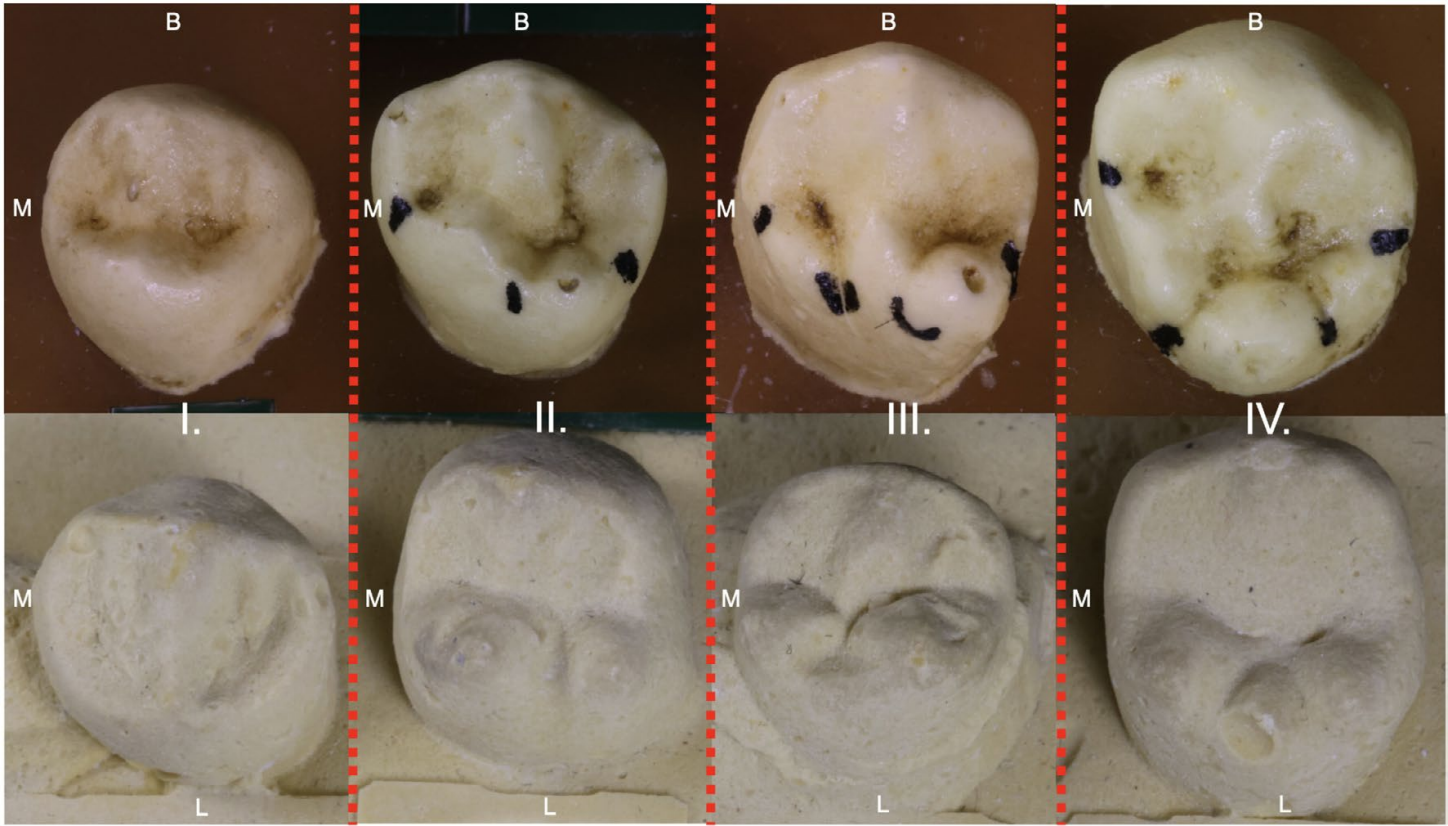
Occlusal view of example lower P3 (top row) and P4 (bottom row) crowns photographed from resin (P3) and dent stone (P4) versions of Turner‐Scott System (ASUDAS) morphology scoring plaques: (I) one lingual cusp—grade 0, (II) two lingual cusps (mesiolingual larger than distolingual)—grade 3, (III) two lingual cusps (mesiolingual smaller than distolingual)—grade 6, and (IV) three lingual cusps—grade 8. Black markings on the P3 exemplars were placed by a previous researcher to emphasize negative topography on the crown surface. B, buccal; L, lingual; M, mesial.

Human mandibular premolars comprise a unique transitional tooth class. The third premolar (P_3_) is characterized by a more caniniform morphology, whereas the fourth premolar (P_4_) is more molariform (White et al. 1991; Krenn et al. 2019). They are composed of one large buccal cusp and one to three (or more) smaller lingual cusps (Türp and Alt 1998; Kocsis et al. 2002; Nelson and Ash 2014) (Figure 1). The enamel knot associated with the buccal cusp forms first, followed by enamel knots for the lingual cusps in a mesial to distal order (Scott 1892; Swindler 2002). This general progression of cusp development is further supported by morphological observation based on expression grades outlined in the Turner‐Scott System (ASUDAS) (Turner et al. 1991). Per Scott and Irish, “When there are accessory cusps, they are usually smaller and distal to the larger mesial lingual cusp” (Scott and Irish 2017, 161). Our PCM‐derived expectations are predicated upon this proposed chronology of crown morphogenesis (see below).

P_3_s are smaller in total crown area than P_4_s (Türp and Alt 1998). P_3_ has a markedly taller buccal cusp than that observed in P_4_ and typically possesses one to two short lingual cusps (Khraisat et al. 2013; Nelson and Ash 2014; Krenn et al. 2019). Three lingual cusped P_3_s can develop but are rare (Nayak et al. 2013). The height disparity between the buccal and lingual cusps is great enough that the lingual cusps often fall well below the occlusal plane as projected from their buccal neighbor (Türp and Alt 1998; Nelson and Ash 2014). P_3_s generally have an elliptical occlusal outline, with the long axis oriented buccolingually (Krenn et al. 2019). They are considered the more morphologically variable tooth of the premolar class, due to their broad anatomical range in cusp number and form (Kraus et al. 1969; Türp and Alt 1998; Krenn et al. 2019).

P_4_s, however, are the more developmentally variable, as they are more likely to be congenitally absent (Kraus et al. 1969; Türp and Alt 1998; Krenn et al. 2019). Two lingual cusped P_4_s are most common, but lingual cusp number can range from one to three in these teeth. While the buccal cusp in P_4_ is large in area, it is of relatively similar height to the lingual cusps (White et al. 1991; Nelson and Ash 2014; Krenn et al. 2019). One lingual cusped P_4_s have a circular occlusal outline, but their crowns become squarer and more molariform as lingual cusp number increases (Nelson and Ash 2014; Krenn et al. 2019).

Compared to human molars, lower premolars possess marked variation in cusp height, which suggests consideration of inhibitory zoning along a z‐dimension is essential to their exploration within a PCM framework. Compared to seal molars, they are multidimensional in cusp configuration, although late‐forming accessory cusps tend to occur only on the lingual surface. Thus, human premolars, with their intermediate morphology, represent an interesting PCM case study.

## 2 Aims and Hypotheses

The aim of this research is to examine the degree to which human mandibular premolar variation conforms to predictions derived from the PCM. A secondary goal is to explore the role of cusp height, as most studies of human occlusal crowns have focused on intercusp spacing measured in only two dimensions. We expected that:

1. Lingual cusp number would be greater in teeth with absolutely larger crowns. This is because a larger tooth germ has more cells and requires a longer period of IEE growth, favoring the formation of more enamel knots and associated accessory cusps (Skinner and Gunz 2010).
2. Lingual cusp number would be greater in teeth with smaller RICDs between earlier forming cusps than in teeth with larger RICDS between earlier forming cusps. Small RICDs are a product of limited inhibitory zones around early‐forming enamel knots (as approximated by cusp tips on the crown surface), which provide space and opportunity for the development of later‐forming cusps (Jernvall 2000; Hunter et al. 2010).
3. Teeth with greater differences in cusp height (i.e., P_3_s) would have fewer lingual cusps than teeth with smaller differences in cusp height (i.e., P_4_s). This is because 3D intercusp dimensions incorporate height, another axis along which inhibitors may act, as originally outlined in Jernvall (2000).
4. Teeth with smaller relative areas of earlier forming cusps (*relative cusp area—*RCA) would have more lingual cusps than teeth with larger RCAs of earlier forming cusps. Small RCAs are a product of constrained inhibitory zones around early‐forming enamel knots, which, again, provide space and opportunity for the development of later‐forming cusps (Jernvall 2000; Hunter et al. 2010).

## 3 Materials and Methods

### 3.1 Dental Cast Sample

This study dataset included two‐dimensional (2D) images and three‐dimensional (3D) scans of anonymized stone casts representing participants of the Harvard Solomon Islands Project (HSIP 1966–1968). Participants were identified in HSIP records as members of Kwaio (*n* = 42) and Baegu (*n* = 21) language groups from the Island of Malaita. Scaled photographs and virtual scans of the dental casts were generated as part of the Global Dental Phenomics Project, aimed at digitally curating and preserving this genealogical dental cast collection. The scans were produced using a Medit Identica Blue scanner at a resolution of approximately 21 μm.

The dataset had equal representation of male and female individuals, with sex identified from HSIP records (n male = 32, n female = 31). The minimum age of individuals included in the sample was approximately 10–12 years old, around the time when mandibular premolars erupt (Smith 1991). Pathological and highly worn teeth were eliminated from the sample, which resulted in a maximum individual age of 50 years. Because all materials and records were previously anonymized, this study was deemed exempt from formal IRB review by the University of Arkansas's Office of Research Integrity and Compliance (see Ethics Statement).

### 3.2 Data Collection

**FIGURE 2:**
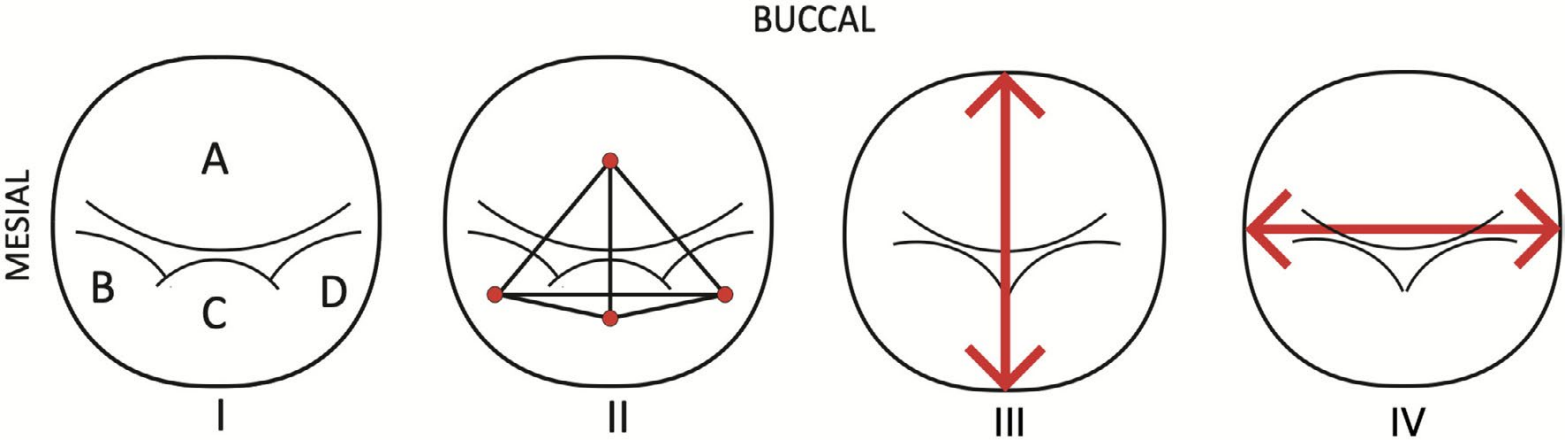
Occlusal view diagrams of three lingual cusped premolars illustrating (I) the naming convention used for cusps and (II) intercusp distance measurements, as well as two lingual cusped premolars illustrating (III) the buccolingual and (IV) mesiodistal measurements used for crown area and for scale when transferring images from MeshLab to ImageJ.

Data were collected from all four mandibular premolars for each individual. MeshLab software was used to quantify lingual cusp number and ICDs using 3D mesh (.ply) files in topographic view (Cignoni et al. 2008). Next, 2D (.jpeg) occlusal surface images were taken of each premolar in MeshLab, with a visible scale measurement extending from the mesial to the distal end of the tooth (Figure 2) (Cignoni et al. 2008). These images were loaded into ImageJ, where the scale measurement was used to translate pixel lengths to millimeters (Schneider et al. 2012). ICDs, crown areas, and lingual cusp counts were collected in ImageJ (Figure 2) (Schneider et al. 2012). Crown area was quantified as the product of maximum buccolingual and mesiodistal diameters. All 2D ICDs were collected in ImageJ to provide x‐ and y‐dimensional measurements (Schneider et al. 2012). All 3D ICDs were collected in MeshLab to account for height differences between cusps along the z‐dimension (Cignoni et al. 2008) (Figure S1). For P_4_s only, cusp area measurements were collected in ImageJ, using the freehand selection/trace tool (Schneider et al. 2012) (Figure S1). Cusp area data were not generated for P_3_s, because cusp margins are not easily distinguishable in these teeth. Raw data are available in Supplemental Data Tables, with individual‐level records available upon request (see Data Availability Statement).

### 3.3 Intra‐Observer Error Study

An intra‐observer error study was performed prior to formal data collection. Data were collected from five dentitions (*n* = 20 premolars) using the protocols described above. Additional crown area measurements (buccolingual and mesiodistal length) were collected in MeshLab (Cignoni et al. 2008). One week later, measurements were recollected. After assessing normality in the dataset using probability plots, a paired two‐tailed *t*‐test was performed in Microsoft Excel (Microsoft Corporation 2018). Intra‐observer error was quantified as less than 3% for all measurements (Table S1). Results show that 3D crown area measurements generated in MeshLab are prone to error. For this reason, MeshLab was only used to collect 3D ICD measurements, which were characterized by high precision (Table S1). All 2D ICD and crown area measurements were highly repeatable when collected in ImageJ. Differences in measurements compared between scoring sessions were both positive and negative, showing no evidence of systematic error associated with data collection protocols (Figures S2–S5).

### 3.4 Statistical Analyses

Absolute dimensions were translated to relative dimensions following Hunter et al. (2010) to account for size variation. To convert ICD measurements to RICDs, raw measurements were divided by the square root of crown area. Cusp area was similarly converted to RCA (relative cusp area), by dividing cusp area by total crown area. A naming convention was created for P_3_ and P_4_ cusps for clarity (Figure 2). The assumption of normality was tested using normal probability plots for lingual cusp number, crown area, RICDs, and RCAs. This assumption was only violated for lingual cusp number, which is an ordinal variable.

#### 3.4.1 Comparing P_3_ and P_4_

Analyses were first conducted using subsamples differentiated by lingual cusp number, comparing P_3_s only with P_4_s that possess the same number of lingual cusps. *F*‐tests of equality of variance were performed for crown area and RICDs. Variances were determined to be statistically equal. A series of independent *t*‐tests were performed to compare crown area and RICDs. For teeth with three lingual cusps, independent *t*‐tests with 1000 bootstrap resampling procedures were performed due to the low number of P_3_s with three lingual cusps. Analyses were then performed to compare P_3_s and P_4_s undifferentiated by lingual cusp number. *F*‐tests of equality of variance were performed for lingual cusp number, crown area, and RICD between cusps A and B (the only cusps consistently present across both P_3_s and P_4_s). Variances were statistically equal for all variables. Independent *t*‐tests were used to compare crown area and RICD from cusp A to cusp B. A Mann–Whitney *U*‐test was performed to compare lingual cusp number between P_3_s and P_4_s. For analyses that used multiple *t*‐tests, Bonferroni corrections were applied to conservatively reduce the probability of type‐I error.

#### 3.4.2 Comparing Antimeres

We then compared left–right pairs of P_3_s and P_4_s. Paired *t*‐tests were performed for crown area and RICDs. For lingual cusp number, a Wilcoxon signed ranks test was performed. To determine the relationship between lingual cusp number and (a) crown area, and (b) RICDs, analysis of variance (ANOVA) tests were run. Independent *t*‐tests with 1000 bootstrap resampling procedures were used to explore the relationship between lingual cusp number and RICD. RICD from cusps A–C and from cusps B–C were used for this analysis, with Bonferroni adjusted alpha‐values.

#### 3.4.3 Testing PCM‐Derived Predictions

Dichotomized generalized linear model (GLM) regressions were completed using lingual cusp number as the response variable. Two models were run for P_3_, with crown area and RICD from cusps A–B as predictors. Four models were run for P_4_, with crown area and RICDs from cusps A–B, cusps A–C, and cusps B–C as predictors.

RCAs were used to further explore variation between two and three lingual cusped P_4_s. Two sets of dichotomized GLM regressions were performed. The first used cusp A (buccal cusp) RCA as a predictor for lingual cusp number, and the second used cusp B (mesiolingual cusp) RCA as a predictor for lingual cusp number. Ordinal least squares (OLS) linear regressions were performed to explore the relationships between RCAs. These models used cusp A RCA as the predictor for the lingual cusp RCAs. A second set of OLS regressions used cusp B RCA as the predictor for cusp C RCA, as well as cusp D RCA in three lingual cusped P_4_s.

As an ad hoc analysis, the angle between cusps A, B, and C was used to examine the effect of early forming cusp position on later forming cusp expression (i.e., cusp D) in two and three lingual cusped premolars. *F*‐tests confirmed equal variances for this variable. Independent *t*‐tests (1,000 bootstrap resampling) were performed. All analyses were conducted in Excel, SPSS, and R (Table S2; Microsoft Corporation 2018; R Core Team 2022; IBM Corporation 2023).

## 4 Results

### 4.1 Morphological Results

Descriptive statistics for P_3_ and P_4_ can be found in Tables S3 and S4 and Figure S6. Of note, average crown area increases for both P_3_ and P_4_ as lingual cusp number increases (Figure S7). In P_4_s, cusp A has the largest average RCA (Figure S8). For both two and three lingual cusped teeth, cusp A comprises approximately 45% of total crown area, on average, while mean RCA decreases across lingual cusps moving distally.

Results indicate significant morphological differences between P_3_s and P_4_s when pooled by lingual cusp number (Table 1). As expected, P_4_s have significantly larger crowns than P_3_s. Results also show a statistically significant difference in lingual cusp number between these neighboring teeth. The P_3_ sample is mostly composed of teeth with two (52%) or one lingual cusp(s) (45%). Only 3% of P_3_s possess three lingual cusps. Two lingual cusped teeth comprise much of the P_4_ sample (63%), with 22% of the sample possessing three lingual cusps, and 15% possessing one (Figure S6). There is no significant difference in 3D RICD from cusps A–B between elements. In the 2D dataset, RICD between A and B differs significantly between P_3_s and P_4_s, with the average RICD for P_4_ exceeding that of P_3_.

**TABLE 1:** Results of morphometric analyses comparing P_3_ and P_4_.

Statistical Test	Variable	*p* ^a^	*α* ^b^	Test Statistic^c^	df^d^	P_3_ ^e^	P_4_ ^e^
3D Data							
Mann–Whitney *U*	Lingual Cusp Number	**< 0.001**	0.050	5.412^c^	250	1.59	2.07
Independent *t*	RICD A–B	0.190	0.050	1.317	250	0.56	0.58
2D Data							
Mann–Whitney *U*	Lingual Cusp Number	**< 0.001**	0.050	5.412^c^	250	1.59	2.07
Independent *t*	Crown Area	**< 0.001**	0.025	5.445	250	60.04	65.29
	RICD A–B	**< 0.001**	0.025	5.397	250	0.52	0.57

^a^ Statistically significant results are bolded with reference to the noted alpha value.

^b^ Overall alpha value is 0.05, unless adjusted using a Bonferroni correction.

^c^ This value is the *t*‐statistic (*t*‐test) or *z*‐score (Mann–Whitney *U*‐test equivalent to the *t*‐statistic).

^d^ Degrees of freedom.

^e^ Mean value for listed element.

There are significant differences between P_3_s and P_4_s in crown area and RICD when comparing teeth with the same number of lingual cusps (Table 2). In the 2D dataset, RICD from A to B significantly differs between P_3_s and P_4_s, in both one and two lingual cusped teeth. Crown area and 2D/3D RICD from B to C significantly differ between two lingual cusped P_3_s and P_4_s. Neighboring premolars with three lingual cusps differ significantly in 3D RICD from A to D, while three lingual cusped metameres differ in 2D RICD from A to C and B to C, and 3D RICD from A to C (*p* < 0.05), these results are not statistically significant with application of a conservative Bonferroni adjusted α‐value to account for type‐I error. For all significant comparisons (except 3D RICD from A to D), P_3_s exhibit smaller mean relative dimensions than P_4_s.

**TABLE 2:** Results of morphometric analyses comparing P_3_ and P_4_ by lingual cusp number.

Lingual Cusp Number	Variable	*p* ^a^	*α* ^b^	*t*‐statistic	df^c^	P_3_ ^d^	P_4_ ^d^
3D Data							
1	RICD A–B	0.31	0.0500	1.02	73	0.56	0.58
2	RICD A–B	0.18	0.0170	1.35	143	0.56	0.58
	RICD A–C	0.09	0.0170	1.73	143	0.67	0.65
	RICD B–C	**< 0.001**	0.0170	8.26	143	0.40	0.51
3	RICD A–B	0.20	0.0083	1.70	30	0.62	0.56
	RICD A–C	0.03	0.0083	1.38	30	0.61	0.65
	RICD B–C	0.16	0.0083	1.69	30	0.31	0.36
	RICD A–D	**< 0.005**	0.0083	2.39	30	0.67	0.61
	RICD B–D	0.77	0.0083	0.79	30	0.57	0.59
	RICD C–D	0.54	0.0083	0.67	30	0.28	0.30
2D Data							
1	Crown Area	0.07	0.0250	1.81	73	59.31	62.80
	RICD A–B	**< 0.01**	0.0250	2.85	73	0.52	0.57
2	Crown Area	**< 0.001**	0.0130	3.85	143	60.68	65.45
	RICD A–B	**< 0.001**	0.0130	4.95	143	0.52	0.58
	RICD A–C	0.11	0.0130	1.63	143	0.61	0.62
	RICD B–C	**< 0.001**	0.0130	8.83	143	0.39	0.52
3	Crown Area	0.07	0.0071	1.80	30	62.40	66.52
	RICD A–B	0.71	0.0071	0.34	30	0.55	0.54
	RICD A–C	0.02	0.0071	1.85	30	0.56	0.62
	RICD B–C	0.02	0.0071	2.04	30	0.30	0.37
	RICD A–D	0.48	0.0071	0.53	30	0.63	0.57
	RICD B–D	0.10	0.0071	1.61	30	0.60	0.60
	RICD C–D	0.96	0.0071	0.54	30	0.33	0.31

^a^ Statistically significant results are bolded with reference to the noted alpha value.

^b^ Overall alpha value of 0.05 was adjusted using a Bonferroni correction.

^c^ Degrees of freedom.

^d^ Mean value for listed element.

Antimeric comparisons indicate no significant differences in lingual cusp number, crown area, or RICD between left and right premolar pairs in the 2D or 3D datasets (Table S5).

### 4.2 ANOVA and *T*‐Test Results

**FIGURE 3:**
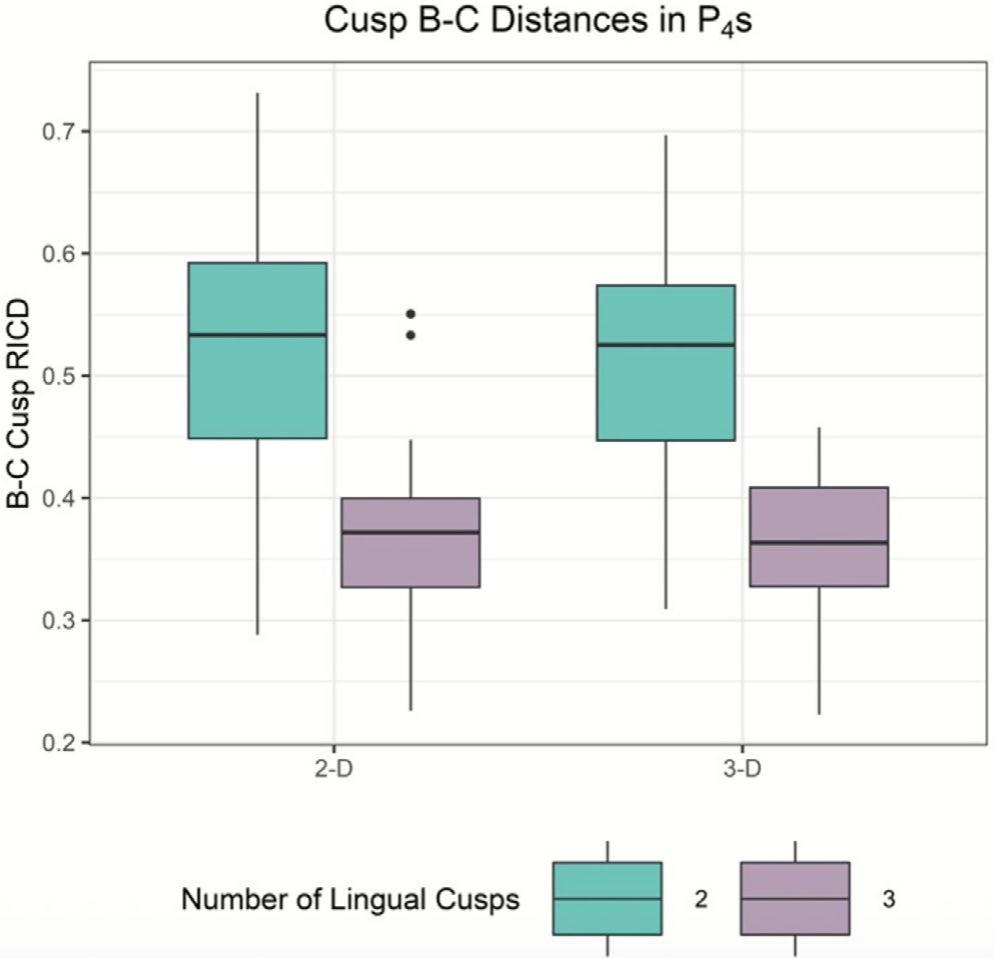
Boxplot showing B–C RICD by lingual cusp number in P_4_s. The boxes on the left reflect the 2D ImageJ dataset; the boxes on the right reflect the 3D MeshLab dataset.

**FIGURE 4:**
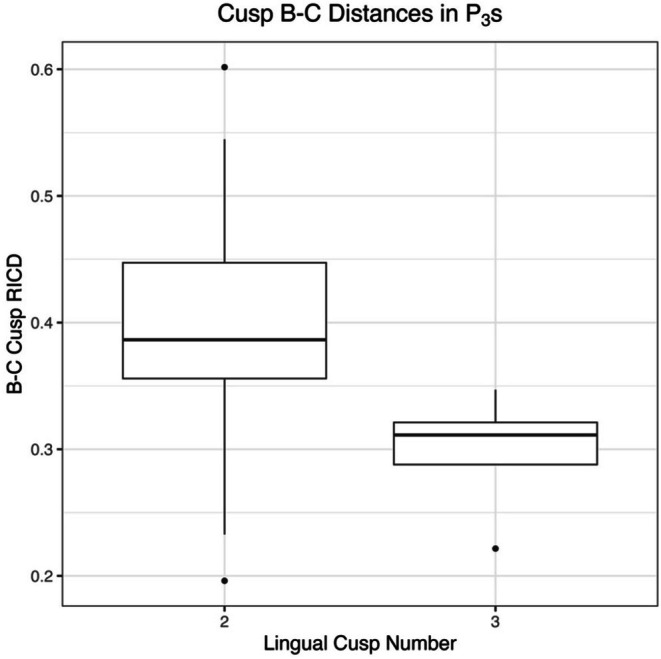
Boxplot showing B–C RICD by lingual cusp number in P_3_s using the 2D ImageJ dataset.

**FIGURE 5:**
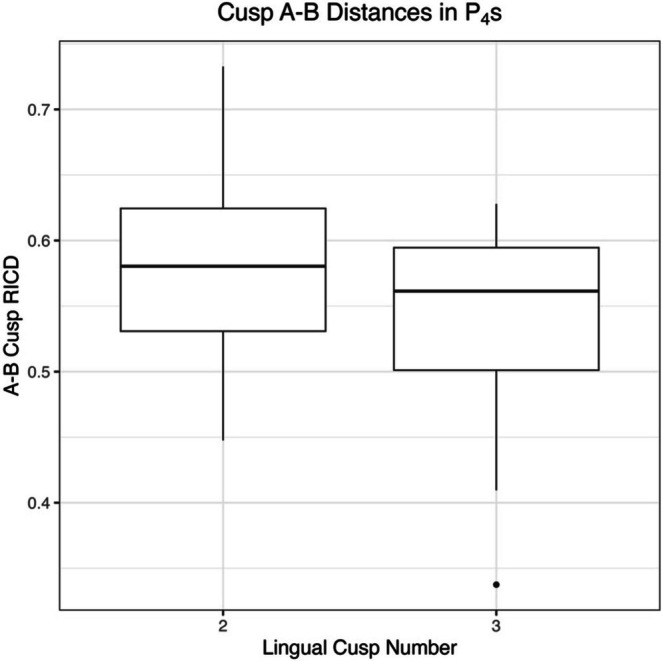
Boxplot showing A–B RICD by lingual cusp number in P_4_s using the 2D ImageJ dataset.

ANOVA results indicate no significant relationship for crown area and lingual cusp number (Table 3). For P_4_, there is a significant relationship between lingual cusp number and RICD from B to C, with RICD decreasing as lingual cusp number increases (Figure 3). This is seen in both the 2D and 3D datasets. For P_3_, a significant relationship exists between lingual cusp number and 2D RICD from B to C, again with RICD decreasing as lingual cusp number increases (Figure 4). Crown area and RICDs do not differ between one and two lingual cusped premolars (for both P_3_s and P_4_s, in both 2D and 3D). However, results indicate significant differences between two and three lingual cusped P_4_s in 2D RICD from cusps A–B (Table 3, Figure 5).

**TABLE 3:** ANOVA results.

Dataset	Variable	*p* ^a^	*F*‐statistic	df^b^
P_3_ Data				
2D (ImageJ)	Crown Area	0.568	0.569	125
	RICD A–B	0.649	0.433	125
	RICD A–C	0.278	1.194	69
	RICD B–C	**< 0.050**	6.359	69
3D (MeshLab)	RICD A–B	0.541	0.617	125
	RICD A–C	0.093	2.465	69
	RICD B–C	0.174	1.794	69
P_4_ Data				
2D (ImageJ)	Crown Area	0.287	1.260	125
	RICD A–B	0.744	0.297	125
	RICD A–C	0.877	0.024	69
	RICD B–C	**< 0.001**	56.434	69
3D (MeshLab)	RICD A–B	0.601	0.512	125
	RICD A–C	0.585	0.300	69
	RICD B–C	**< 0.001**	61.337	69
P_3_ Data (One Lingual Cusped Teeth Omitted)				
2D (ImageJ)	Crown Area	0.643	0.216	69
	RICD A–B	0.432	0.624	69
3D (MeshLab)	RICD A–B	0.148	2.145	69
P_4_ Data (One Lingual Cusped Teeth Omitted)				
2D (ImageJ)	Crown Area	0.534	0.390	69
	RICD A–B	**< 0.010**	7.391	69
3D (MeshLab)	RICD A–B	0.297	1.099	69

^a^ Statistically significant results (*p* < 0.05) are bolded.

^b^ Degrees of freedom.

**FIGURE 6:**
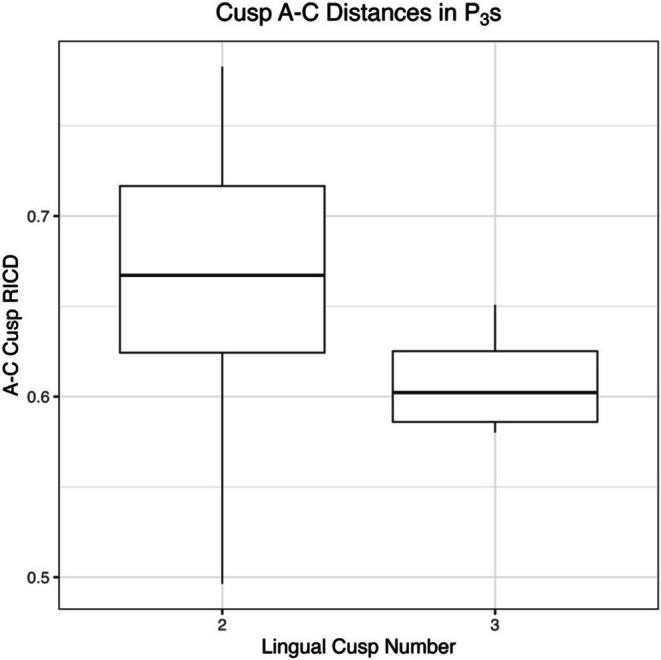
Boxplot showing A–C RICD by lingual cusp number in P_3_s using the 3D MeshLab dataset.

*T*‐test results comparing RICD from cusps A–C and B–C are presented in Table 4 and are consistent with and without the application of bootstrapping procedures. RICD from cusps B–C differs significantly across premolars of distinct lingual cusp number, with two lingual cusped premolars exhibiting greater RICD on average than three lingual cusped premolars. These results are consistent in the 2D dataset for P_3_s, and in both the 2D and 3D datasets for P_4_s. Of note, the *p*‐value for the P_3_ 3D RICD B–C model is less than 0.05, but greater than the Bonferroni corrected α of 0.025. The 3D RICD from A to C in P_3_s also varies as a function of lingual cusp number, with two lingual cusped teeth exhibiting greater RICD, on average, however these results are not statistically significant with application of a Bonferroni α correction (Figure 6).

**TABLE 4:** Independent *t*‐test results with bootstrap resampling.

Dataset	Variable	*p* ^a^	α^b^	*t*‐statistic	df^c^
P_3_ Data					
2D (ImageJ)	RICD A–C	0.278	0.025	1.09	69
	RICD B–C	**0.014**	0.025	2.52	69
3D (MeshLab)	RICD A–C	0.026	0.025	3.24	69
	RICD B–C	0.030	0.025	2.22	69
P_4_ Data					
2D (ImageJ)	RICD A–C	0.609	0.025	0.512	105
	RICD B–C	**< 0.001**	0.025	7.704	105
3D (MeshLab)	RICD A–C	0.831	0.025	0.214	105
	RICD B–C	**< 0.001**	0.025	8.424	105

^a^ Statistically significant results are bolded with reference to the noted alpha value.

^b^ The overall alpha value of 0.05 was recalculated using a Bonferroni correction.

^c^ Degrees of freedom.

### 4.3 Regression Results

**FIGURE 7:**
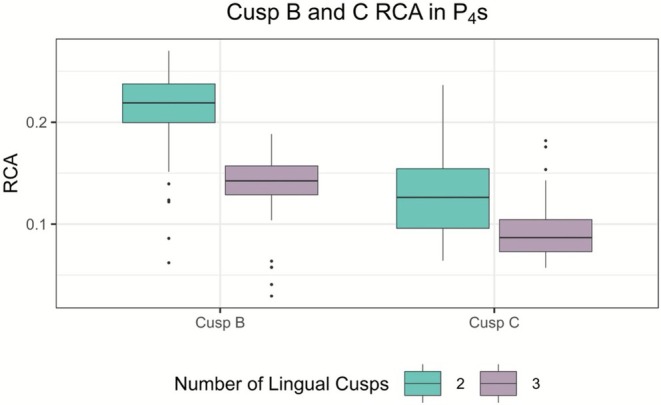
Comparative boxplots showing RCAs for cusp B (left) and cusp C (right) by lingual cusp number.

GLM regression results can be found in Table 5. Again, no significant relationship is indicated for lingual cusp number and crown area. However, both 2D and 3D results show a strong link between lingual cusp number and RICD from B to C. For the P_4_ sample, results indicate a significant relationship between lingual cusp number and 2D RICD from A to B. These results are consistent with ANOVA and independent *t*‐test output. Results also reveal RCAs of A, B, and C to be significant predictors of lingual cusp number in P_4_ (Table 5). Based on odds ratio values, RCA of B and C appear to be stronger predictors of lingual cusp number than that of A. Interestingly, RCA of the buccal cusp (A) is smaller in P_4_s with two lingual cusps than in P_4_s with three lingual cusps, while the opposite is true for RCAs of both B and C, where the RCAs are larger in P_4_s with two lingual cusps than in P_4_s with three lingual cusps (Figure 7).

**TABLE 5:** GLM regression results for models with lingual cusp number as the response variable.

Dataset	Predictor	*p* ^a^	*z*‐score	df^b^	Odds ratio
P_3_ Data					
2D (ImageJ)	Crown Area	0.459	0.740	120	0.35
	RICD A–B	0.754	0.314	120	0.77
3D (MeshLab)	RICD A–B	0.871	0.163	120	1.00
P_4_ Data					
2D (ImageJ)	Crown Area	0.530	0.628	105	0.11
	RICD A–B	**< 0.050**	2.548	105	42.30
	RICD A–C	0.606	0.516	105	1.25
	RICD B–C	**< 0.001**	4.461	105	1.81 × 10^3^
3D (MeshLab)	RICD A–B	0.295	1.047	105	2.57
	RICD A–C	0.829	0.216	105	0.21
	RICD B–C	**< 0.001**	4.794	105	1.19 × 10^4^
	Cusp A RCA	**< 0.050**	2.444	105	3.61 × 10^−4^
	Cusp B RCA	**< 0.001**	4.951	105	1.31 × 10^3^
	Cusp C RCA	**< 0.001**	3.495	105	7.48

^a^ Statistically significant results (*p* < 0.05) are bolded.

^b^ Degrees of freedom.

**FIGURE 8:**
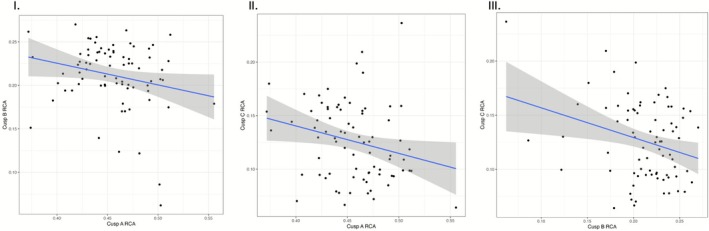
Bivariate plots for cusp RCAs in two lingual cusped P_4_s with regression lines. The gray regions show the 95% confidence intervals. (I) cusp A and cusp B, (II) cusp A and cusp C, and (III) cusp B and cusp C.

**FIGURE 9:**
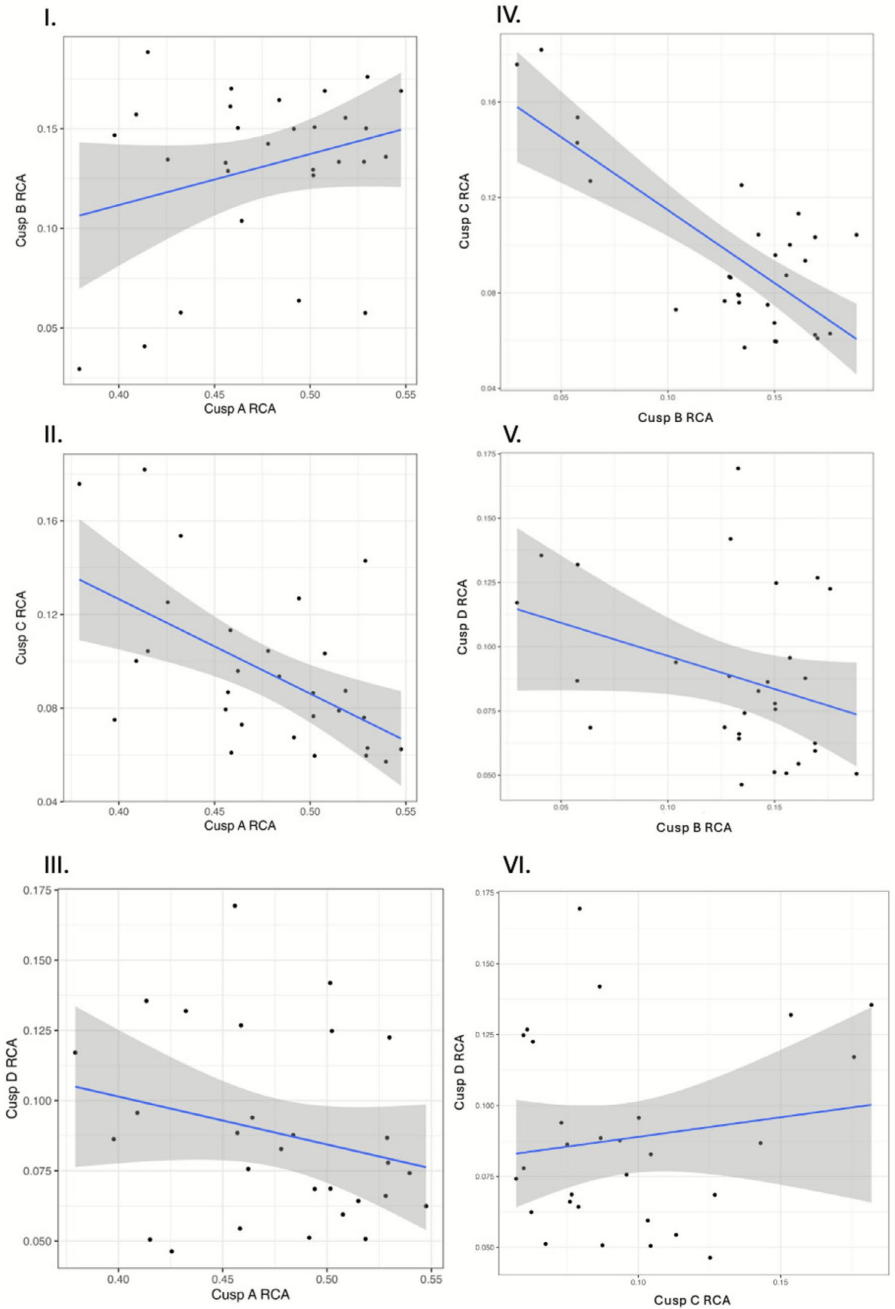
Bivariate plots for cusp RCAs in three lingual cusped P_4_s with regression lines. The gray regions show the 95% confidence intervals. (I) cusp A and cusp B, (II) cusp A and cusp C, (III) cusp A and cusp D, (IV) cusp B and cusp C, (V) cusp B and cusp D, and (VI) cusp C and cusp D.

OLS regression results are presented in Table 6. All models for two‐cusped teeth are significant, with slopes of approximately −0.250 (Figure 8). However, the *R* ^2^ values are low (< 0.100). In three‐cusped teeth, OLS regression using cusp A RCA as the predictor for cusp C RCA is significant with a slope of −0.404, but the *R* ^2^ value is low (0.280) (Figure 9). The model using cusp B RCA as the predictor for cusp C RCA is also significant, with a slope of −0.602. Of the significant regression models, cusp B RCA as a predictor for cusp C RCA yields the highest *R* ^2^ value at 0.553. Other models for three lingual cusped P_4_s are not statistically significant.

**TABLE 6:** Linear regression results for relative cusp areas.

Predictor Variable	Response Variable	*p* ^a^	*t*‐value	Slope	*R* ^2^
Two Lingual Cusped P_4_s					
Cusp A	Cusp B	**< 0.050**	−2.04	−0.250	0.039
	Cusp C	**< 0.050**	−2.21	−0.256	0.048
Cusp B	Cusp C	**< 0.050**	−2.63	−0.275	0.071
Three Lingual Cusped P_4_s					
Cusp A	Cusp B	0.135	1.54	0.256	0.047
	Cusp C	**< 0.010**	−3.45	−0.404	0.280
	Cusp D	0.199	−1.32	−0.171	0.026
Cusp B	Cusp C	**< 0.001**	−5.98	−0.612	0.553
	Cusp D	0.077	−1.84	−0.258	0.079
Cusp C	Cusp D	0.453	0.76	0.138	0.015

^a^ Statistically significant results (*p* < 0.05) are bolded.

## 5 Discussion

### 5.1 Morphological Comparisons

The results of this study are partially consistent with PCM‐derived predictions surrounding crown configuration and morphology. While the relationship is not statistically significant, as a general trend, crown area increases as lingual cusp number increases following the PCM prediction that a larger tooth germ, and thus a larger developed tooth, will possess more accessory cusps (Jernvall 2000). There is a positive relationship between tooth germ size and developmental duration, with prolonged growth of the IEE favoring the formation of accessory cusps (Jernvall 2000; Jernvall and Jung 2000; Salazar‐Ciudad and Jernvall 2010). Also in accordance with the PCM, relative distance between the earliest forming lingual cusps (B and C) decreases as lingual cusp count increases from two to three. Other RICD measurements among earlier forming cusps (A–B and A–C) variably increase and decrease by cusp number.

For the 3D dataset, this is likely related to the inclusion of cusp A. This buccal cusp accounts for the majority of mandibular premolar cusp height disparity; all lingual cusps are of more similar height. Variation in cusp A height results in disparate RICD measurements across teeth. These inconsistencies can also be attributed to variation in cusp B and C positioning. In one lingual cusped premolars, cusp B is centered on the lingual side of the tooth, but in premolars with multiple lingual cusps, cusp B is more mesially positioned. Similarly, cusp C is more distally positioned in two‐cusped premolars, but when cusp D is present it is more centered. The decrease in P_4_ mean RCA from cusp B to D follows Jernvall's (2000) prediction that later forming cusps (C and D) are smaller and more variable than early forming cusps.

Results of P_3_ and P_4_ comparisons corroborate the notion that human mandibular premolars represent a transitional tooth class. Aligning with PCM‐derived predictions, P_4_, the significantly larger premolar, has a greater number of lingual cusps than P_3_, on average. Because buccal/lingual cusp height disparity is more prominent in P_3_s than in P_4_s, it was expected their RICDs from A to B would differ significantly in the 3D dataset. Interestingly this is not the case; RICD from A to B only significantly differs between P_3_s and P_4_s in the 2D dataset (P_3_ A–B RICD = 0.521; P_4_ A–B RICD = 0.569). In other words, the inclusion of the z‐dimension in cusp distance measures results in P_3_s and P_4_s appearing more similar in configuration (P_3_ A–B RICD = 0.564; P_4_ A–B RICD = 0.575). With the inclusion of the z‐dimension, the P_3_ mean substantially increases, due to the greater A–B cusp height disparity in this tooth. The z‐dimension adds enough distance to the P_3_ measurement to make it similar to the P_4_ measurement, obscuring configurational differences between the two. These results are consistent with P_4_s being larger in area and having more molariform morphology and P_3_s being smaller in area with a more caniniform morphology.

P_3_s and P_4_s with two lingual cusps exhibit several significant differences in configuration. Two lingual cusped P_3_s and P_4_s are the only metameres that differ significantly in crown area. This result may drive the significant difference in crown area between P_3_s and P_4_s in the pooled analysis. Surprisingly, one and three lingual cusped P_3_s and P_4_s do not differ significantly in crown area. As crown area is the only raw metric variable in the study (i.e., not adjusted for size), these results may be biased by sample composition, as the sample of three lingual cusped P_3_s is limited (*n* = 4). RICD from cusps A–B differs significantly between metameres with one and two lingual cusps, but in the 2D dataset only.

The RICD from cusps B–C only differs significantly between two lingual cusped P_3_s and P_4_s. This is potentially due to the morphology of lingual cusps in P_3_s. Cusps B and C are considerably more condensed in two lingual cusped P_3_s than in P_4_s, as the buccal cusp takes up more of the total crown area in P_3_s. However, in three lingual cusped teeth, cusps B and C must be closer together in P_4_s to induce the formation of cusp D; therefore, the distance between the mesiolingual cusps is more condensed and similar to P_3_s. This result is consistent across the 2D and 3D datasets, as the heights of cusps B and C are fairly similar. However, RICD from A–D only differs significantly between metameres in the 3D dataset, meaning cusp height plays a major role in differentiating this distance between premolars. With the inclusion of the z‐dimension, the P_3_ mean increases due to the height of buccal cusp A.

The lack of significant difference between antimeres in both P_3_s and P_4_s indicates high levels of symmetry among these teeth. Fluctuating asymmetry in dental crown morphology suggests some degree of environmental intervention in enamel knot formation (Townsend et al. 2003; Hunter et al. 2010; Paul et al. 2023). Bilateral symmetry might suggest strong developmental stability of premolar crown configuration for this sample.

### 5.2 Analytical Discussion

Some results of the cusp distance analyses align with the PCM. For all significant models, RICDs are smaller in teeth with more lingual cusps. However, P_3_ and P_4_ lingual cusp numbers are dependent on different RICD measurements, which reflect the distinct morphologies of these teeth. Three lingual cusped P_3_s are rarer and their lingual cusps are generally more condensed due to their prominent buccal cusp. Inhibitory zones in P_3_s may be smaller than those in P_4_s on average, resulting in the secondary enamel knots forming closer together. Likewise, the cusp‐activating signaling protein, Fgf4, may be genetically influenced to induce secondary enamel knots closer together in P_3_s than in P_4_s.

Significant results linking cusp spacing to lingual cusp number in P_4_ involve cusp B. Thus, the inhibitory zone around the mesiolingual enamel knot seems to have the greatest impact on P_4_'s ultimate crown morphology. This is expected, as cusp B is the earliest lingual cusp to form. This chronology is corroborated by our individual‐level z‐coordinate data that shows it to be the tallest of lingual cusps in premolars with multiple lingual cusps in all but 4% of cases. Its zone of inhibition appears to have a disproportionate influence on the possible formation of later forming distolingual cusps. Further exemplifying the “cascade” effect outlined in the PCM, the inhibitory zone of cusp C also strongly influences the development and expression of its immediate distal neighbor, cusp D, here presumed to be later forming.

For P_3_s, results suggest an association between lingual cusp number and (a) 2D RICD from B to C, and (b) 3D RICD from A to C. While these relationships are not statistically significant after adjusting for potential type‐I error, we note that Bonferroni correction can be overly conservative. The inhibitory zones of cusps B and C seem to have the strongest influence on the formation and expression of a third lingual cusp in P_3_s. When the z‐dimension is considered, cusp C's relative distance from cusp B and cusp A is implicated. It appears cusp C's inhibitory zone is more influential than cusp B's on the formation of a third lingual cusp. This represents a morphogenetic divergence between P_3_s and P_4_s, where in P_4_s the inhibitory zone of cusp B has the greatest influence.

In comparisons of one and two lingual cusped teeth, neither crown area nor RICD from A–B are significantly linked to lingual cusp number. It is unclear, epigenetically, what is regulating the formation of a second lingual cusp in mandibular premolars. Because distance between cusps A and B does not markedly differ, this phenomenon is likely unrelated to inhibitory zone size. However, with the addition of other lingual cusps, the positioning of cusp B shifts from central‐lingual to mesiolingual. This suggests that the initiation of cusp B's enamel knot in a more mesial position is driving the formation of extra lingual cusps. In some individuals, primary enamel knot signaling directs a secondary enamel knot to arise central‐lingually, while in others, they direct the secondary enamel knot to arise mesiolingually. An ad‐hoc exploration of mesiolingual (A–B–C) cusp angle supported this interpretation^1^ (Tables S3 and S6). Three lingual cusped premolars exhibit significantly more acute A–B–C angles (P_3_s = 25.15°; P_4_s = 27.73°) than two lingual cusped premolars (P_3_s = 33.84°; P_4_s = 42.65°).

Relative areas of cusps A, B, and C are all significant predictors of lingual cusp number in two and three lingual cusped P_4_s. This was an unexpected result for cusp A, as it composes roughly half of total P_4_ crown area, regardless of lingual cusp number. Not only is it a significant predictor of lingual cusp number, but it is also larger in teeth with three lingual cusps than in teeth with two lingual cusps. This is in opposition to a prediction yielded by the PCM, that a smaller inhibitory zone for cusp A will allow space for later‐forming accessory lingual cusps. Of note, the sample size for three lingual cusped P_4_s (*n* = 28) was small. Relationships between lingual cusp number and RCAs of B and C are more in line with PCM‐derived expectations. The smaller cusps B and C are, the more likely it is an extra lingual cusp will form. These models had much higher odds ratio values than those involving cusp A, supporting the claim that inhibitory zones of cusps B and C are most influential in determining the formation of a third lingual cusp. Linear regression results, while significant, have low *R* ^2^ values, denoting predictor and response variables are correlated, but their relationship fails to adequately explain observed variation. It is possible that Edar, the protein associated with enamel knot size and thus final cusp size, is uniquely expressed for each enamel knot (Tucker et al. 2000).

### 5.3 Differences Between 2D and 3D Analyses

Premolars, especially P_3_s, are characterized by significant cusp height disparity between the buccal and lingual cusps. Overall though, 2D and 3D datasets yield similar results. This indicates 2D and 3D measurements at the crown surface provide a similar picture of crown form in premolars. Inclusion of the z‐dimension does not notably enhance detection of an underlying biological signal, at least not one that conforms to evo‐devo model‐derived expectations. Our findings suggest that the even height of hominin molar cusps and the differential application of 3D distance data do not account for the mixed results yielded by previous PCM studies (Jernvall and Jung 2000; Hlusko et al. 2004; Hunter et al. 2010; Skinner and Gunz 2010; Moormann et al. 2013; Morita et al. 2016; Paul et al. 2017; Clark and Guatelli‐Steinberg 2018; Ortiz et al. 2018).

While these findings provide new insight into the morphogenetic foundations of mandibular premolar variation, this study is not without limitations. First, the sample included few three lingual cusped P_3_s. Future studies may focus on samples that contain greater variation in lingual cusp number. Second, it is possible our models were based on an inaccurate chronology of cusp formation. Documented global trends in human morphology (Scott and Irish 2017) point to a common order of cusp development: (A) buccal cusp, (B) mesiolingual cusp, (C) central lingual cusp (in three lingual cusped teeth), and (D) distolingual cusp. In support of this chronology, inverse individual‐level z‐coordinate data from scans show the buccal cusp to be the tallest in all teeth in our sample. For premolars with multiple lingual cusps, the mesiolingual one was overwhelmingly the tallest (~96%) cusp after its buccal neighbor.

That said, among all three lingual cusped premolars, there are nine cases (~28%) in which the distolingual cusp is taller than the central lingual cusp. We note that these cases all occur in P_4_s and that they are characterized by limited height difference between the middle and distolingual cusps (mean = 0.21 mm). As such, it is possible, in these elements, that both cusps initiate around the same time, as would be expected for a tall Carabelli's cusp on an upper molar (Hunter et al. 2010, 1). Alternatively, this pattern may correspond with a different order of cusp development than the one assumed in our study; in these cases, we would instead expect a greater distance between the mesiolingual and distolingual cusps to accommodate a later‐forming central cusp between them. Yet, in our three cusped P_4_ sample, there is no significant difference in absolute or relative cusp B–D distance that distinguishes teeth with taller central lingual cusps from those with taller distolingual cusps (ICD *p* = 0.80, RICD *p =* 0.69).^2^ In fact, there is no significant difference in any cusp distance between these two subsamples that would clarify the nature and chronology of lingual cusp initiation. For this reason, three lingual cusped premolars represent an important avenue for expanded research, as previous EDJ studies have documented the development of accessory cusps at distinct locations along the mesial and distal marginal ridges of hominid lower premolars (Davies et al. 2019). Ultimately, for lower premolars, it may be the case that “…not all configurations that promote the initiation of accessory cusps have been identified and considered” (Blankenship‐Sefczek et al. 2024, 3).

Finally, RICDs represent measurements taken at the enamel surface rather than at the EDJ. MicroCT studies of EDJ morphology have found that some “cusps” present on the outer enamel surface of quadrate molars do not have associated dentin horns and therefore lack enamel knot precursors (Skinner and Gunz 2010; Ortiz et al. 2017, 2018). This underscores potential issues with using cusp tips as enamel knot proxies in human postcanine teeth, and it is possible some cusps included in this study may not be direct proxies for enamel knots. This is especially a concern for the P_3_ sample. P_3_ lingual cusps, like many accessory cusps, are relatively small and can be difficult to identify, even at the EDJ (Davies et al. 2019). The challenge of distinguishing lingual cusps is greater at the outer enamel surface and, in some cases, greater still in dent stone or plaster dental models—a complication that impacted sample size in the present study.

## 6 Conclusion

This study examined the correspondence of PCM‐derived predictions to observed human mandibular premolar variation. Results suggest significant relationships between crown morphology (i.e., lingual cusp number) and certain RICDs and RCAs. The inhibitory zones of cusps B and C are highly influential in the expression of third lingual cusps in P_3_s and P_4_s. Interestingly, the inhibitory zone of cusp A also impacts the formation and expression of third lingual cusps, despite its relatively constant location and size. Accounting for the *z*‐axis (i.e., height) in teeth without marked cusp height disparities (P_4_s) does not yield results more aligned with PCM‐derived expectations. For teeth with greater cusp height disparities (P_3_s), the *z*‐axis is important for capturing certain cusp relationships—in particular, those between the tall buccal cusp A and the shorter lingual cusps—as well as the corresponding inhibitory zones surrounding their associated enamel knots in three dimensions. Future research should expand analyses to include human samples representing several global populations, especially those with varying frequencies of three or more lingual cusped premolars, and explore the potential for alternative sequential orders of cusp initiation in these teeth.

## Author Contributions

**Molly Militello:** conceptualization, investigation, writing – original draft, methodology, visualization, formal analysis, validation, data curation. **Dori E. Kenessey:** formal analysis, methodology, supervision. **Christopher M. Stojanowski:** conceptualization, data curation, resources, writing – review and editing. **Kathleen S. Paul:** conceptualization, writing – review and editing, project administration, data curation, resources, supervision, investigation.

## Funding

This work was supported by the National Science Foundation (BCS‐1750089).

## Ethics Statement

This study was deemed exempt from IRB review by the University of Arkansas' Office of Research Integrity and Compliance and the IRB of Arizona State University pursuant to Federal Regulation 45CFR46(4)—Study 00007452.

## Supporting information


**Data Table 1** Left P_3_s 2D ImageJ dataset.
**Data Table 2**. Right P_3_s 2D ImageJ dataset.
**Data Table 3**. Left P_4_s 2D ImageJ dataset.
**Data Table 4**. Right P_4_s 2D ImageJ dataset.
**Data Table 5**. Left P_3_s 3D MeshLab dataset.
**Data Table 6**. Right P_3_s 3D MeshLab dataset.
**Data Table 7**. Left P_4_s 3D MeshLab dataset.
**Data Table 8**. Right P_4_s 3D MeshLab dataset.
**Data Table 9**. Cusp area for two lingual cusped P_4_s.
**Data Table 10**. Cusp area for three lingual cusped P_4_s.

**Table S1:** Results of the paired *t*‐tests from the intra‐observer error analysis.
**Table S2:** Software used for each statistical analysis.
**Table S3:** Descriptive statistics for P3 and P4 variables by lingual cusp number.
**Table S4:** Descriptive statistics for lingual cusp number in P3 and P4.
**Table S5:** Results of the antimeric analyses for left and right elements.
**Table S6:** Independent t‐test results comparing mean A‐B‐C cusp angle in two and three lingual cusped teeth (bootstrap resampling applied).
**Figure S1:** Occlusal views of a P4 crown with topographic overlay in MeshLab (I) and ImageJ (II). (I) Cusp tip locations are approximated and marked by black dots with reference to the curvature map and regional maxima along the z‐axis with adjustments made for wear or alternative cusp morphology as viewed from the original images. Solid black lines between cusp tips represent intercusp distances. (II) Cusp areas traced using ImageJ's freehand selection/trace tool, with shading used to distinguish the buccal (blue), mesiolingual (yellow), and distolingual (red) cusps.
**Figure S2:** Bar graph illustrating raw measurements from the intra‐observer error study for ICDs in MeshLab.
**Figure S3:** Bar graphs illustrating raw measurements from the intra‐observer error study for crown area in MeshLab. Top (A) is mesial‐distal length, and bottom (B) is buccal‐lingual length.
**Figure S4:** Bar graph illustrating raw measurements from the intra‐observer error study for ICDs in ImageJ.
**Figure S5:** Bar graphs illustrating raw measurements from the intra‐observer error study for crown area in ImageJ. Top (A) is mesial‐distal length, and bottom (B) is buccal‐lingual length.
**Figure S6:** Pie charts comparing (A) P3 sample by lingual cusp number, and (B) P4 sample by lingual cusp number.
**Figure S7:** Crown area by lingual cusp number for (A) P3s, and (B) P4s.
**Figure S8:** Pie charts comparing (A) two lingual cusped P4 by average RCA, and (B) three lingual cusped P4 by average RCA.

## Data Availability

The data that supports the findings of this study are available in the Supporting Information of this article.

## Endnotes

^1^
As part of an ad hoc analysis, angular measurements between the mesial‐most lingual cusp and distal neighboring cusp were collected in ImageJ for two and three lingual cusped teeth (Cignoni et al. 2008). Descriptive statistics for A–B–C cusp angle can be found in Table S3. P_3_s and P_4_s with two lingual cusps have a larger mean A–B–C cusp angle than P_3_s and P_4_s with three lingual cusps. For all continuous measurements, standard deviation is one order of magnitude lower than the average measurement. T‐test results comparing A‐B‐C cusp angle in two and three lingual cusped teeth are presented in Table S6. A‐B‐C cusp angle differ significantly between two and three lingual cusped premolars for both P_3_s and P_4_s, with two lingual cusped premolars exhibiting a greater angle on average.

^2^
These ad‐hoc analysis were conducted using 2D distance measurements generated in ImageJ.
