# Supplementary material for: The Patterning Cascade Model and Human Mandibular Premolar Variation
Source: Am J Biol Anthropol. 2025 Dec 9;188(4):e70178. doi: 10.1002/ajpa.70178 (PMC12687927; doi:10.1002/ajpa.70178)
Supplement: Supplementary file 1 — Data Table 1 Left P3s 2D ImageJ dataset. Data Table 2. Right P3s 2D ImageJ dataset. Data Table 3. Left P4s 2D ImageJ dataset. Data Table 4. Right P4s 2D ImageJ dataset. Data Table 5. Left P3s 3D MeshLab dataset. Data Table 6. Right P3s 3D MeshLab dataset. Data Table 7. Left P4s 3D MeshLab dataset. Data Table 8. Right P4s 3D MeshLab dataset. Data Table 9. Cusp area for two lingual cusped P4s. Data Table 10. Cusp area for three lingual cusped P4s. [file AJPA-188-e70178-s002.docx]

**RAW DATA**

Data Table 1. Left P_3_s 2D ImageJ Dataset

| Lingual Cusp Number | ICD A-B (mm) | ICD A-C (mm) | ICD B-C (mm) | ICD C-D (mm) | ICD A-C (mm) | ICD B-D (mm) | MD^a^ (mm) | BL^b^ (mm) |
| --- | --- | --- | --- | --- | --- | --- | --- | --- |
| 1 | 5.067 | - | - | - | - | - | 6.953 | 8.473 |
| 1 | 4.247 | - | - | - | - | - | 8.264 | 8.276 |
| 1 | 3.929 | - | - | - | - | - | 7.276 | 8.021 |
| 1 | 3.507 | - | - | - | - | - | 6.858 | 7.907 |
| 1 | 3.433 | - | - | - | - | - | 6.693 | 8.878 |
| 1 | 5.028 | - | - | - | - | - | 7.730 | 9.527 |
| 1 | 3.274 | - | - | - | - | - | 7.810 | 8.086 |
| 1 | 3.977 | - | - | - | - | - | 7.170 | 8.580 |
| 1 | 3.200 | - | - | - | - | - | 6.989 | 8.135 |
| 1 | 4.192 | - | - | - | - | - | 7.253 | 9.541 |
| 1 | 3.950 | - | - | - | - | - | 6.08 | 8.153 |
| 1 | 4.534 | - | - | - | - | - | 7.380 | 8.189 |
| 1 | 3.580 | - | - | - | - | - | 7.314 | 8.586 |
| 1 | 3.323 | - | - | - | - | - | 7.373 | 9.315 |
| 1 | 3.777 | - | - | - | - | - | 7.234 | 7.544 |
| 1 | 4.054 | - | - | - | - | - | 6.362 | 7.504 |
| 1 | 3.521 | - | - | - | - | - | 7.711 | 8.159 |
| 1 | 4.170 | - | - | - | - | - | 7.742 | 7.336 |
| 1 | 3.774 | - | - | - | - | - | 6.597 | 7.884 |
| 1 | 4.286 | - | - | - | - | - | 6.668 | 8.079 |
| 1 | 3.341 | - | - | - | - | - | 7.126 | 8.262 |
| 1 | 4.406 | - | - | - | - | - | 7.612 | 7.214 |
| 1 | 3.329 | - | - | - | - | - | 7.083 | 7.048 |
| 1 | 3.247 | - | - | - | - | - | 5.976 | 7.118 |
| 1 | 4.882 | - | - | - | - | - | 7.325 | 8.493 |
| 1 | 5.178 | - | - | - | - | - | 8.005 | 9.044 |
| 2 | 3.843 | 4.322 | 3.046 | - | - | - | 7.848 | 9.477 |
| 2 | 4.471 | 5.182 | 3.649 | - | - | - | 7.225 | 8.919 |
| 2 | 4.573 | 4.748 | 3.503 | - | - | - | 7.644 | 8.918 |
| 2 | 4.263 | 4.275 | 3.424 | - | - | - | 6.404 | 8.185 |
| 2 | 3.742 | 4.404 | 3.394 | - | - | - | 7.496 | 8.399 |
| 2 | 3.601 | 4.981 | 2.989 | - | - | - | 7.646 | 8.424 |
| 2 | 3.477 | 5.401 | 4.385 | - | - | - | 7.031 | 9.216 |
| 2 | 5.154 | 6.140 | 2.951 | - | - | - | 7.063 | 8.696 |
| 2 | 3.741 | 4.480 | 3.146 | - | - | - | 6.710 | 8.309 |
| 2 | 4.255 | 5.001 | 2.793 | - | - | - | 7.022 | 7.721 |
| 2 | 4.757 | 4.649 | 3.212 | - | - | - | 7.589 | 9.557 |
| 2 | 4.800 | 5.606 | 3.469 | - | - | - | 8.167 | 9.166 |

Data Table 1 Continued.

| Lingual Cusp Number | ICD A-B (mm) | ICD A-C (mm) | ICD B-C (mm) | ICD C-D (mm) | ICD A-C (mm) | ICD B-D (mm) | MD^a^ (mm) | BL^b^ (mm) |
| --- | --- | --- | --- | --- | --- | --- | --- | --- |
| 2 | 4.376 | 5.849 | 4.197 | - | - | - | 8.033 | 9.786 |
| 2 | 4.666 | 4.766 | 2.747 | - | - | - | 6.780 | 8.549 |
| 2 | 4.354 | 5.373 | 4.097 | - | - | - | 6.708 | 9.494 |
| 2 | 4.706 | 4.722 | 2.326 | - | - | - | 6.411 | 8.402 |
| 2 | 4.135 | 4.501 | 2.657 | - | - | - | 5.690 | 8.169 |
| 2 | 3.761 | 5.364 | 3.359 | - | - | - | 7.025 | 9.085 |
| 2 | 3.942 | 3.925 | 2.020 | - | - | - | 7.091 | 8.055 |
| 2 | 3.883 | 4.122 | 2.660 | - | - | - | 7.861 | 9.656 |
| 2 | 3.418 | 4.378 | 2.657 | - | - | - | 6.737 | 8.560 |
| 2 | 4.438 | 3.994 | 2.853 | - | - | - | 6.864 | 8.591 |
| 2 | 2.937 | 3.808 | 2.815 | - | - | - | 7.697 | 7.662 |
| 2 | 4.016 | 4.577 | 1.883 | - | - | - | 7.384 | 8.870 |
| 2 | 4.013 | 3.764 | 3.152 | - | - | - | 6.215 | 7.673 |
| 2 | 3.215 | 2.941 | 2.984 | - | - | - | 6.222 | 8.226 |
| 2 | 4.692 | 5.005 | 2.264 | - | - | - | 6.859 | 8.437 |
| 2 | 3.610 | 4.218 | 2.192 | - | - | - | 7.242 | 8.578 |
| 2 | 3.544 | 4.625 | 3.359 | - | - | - | 7.440 | 8.361 |
| 2 | 3.987 | 4.317 | 2.767 | - | - | - | 7.292 | 7.645 |
| 2 | 4.270 | 4.723 | 2.783 | - | - | - | 7.283 | 8.531 |
| 2 | 3.803 | 4.340 | 2.809 | - | - | - | 6.796 | 7.564 |
| 2 | 3.537 | 4.028 | 2.931 | - | - | - | 6.316 | 7.280 |
| 2 | 3.705 | 5.333 | 2.453 | - | - | - | 7.328 | 8.640 |
| 3 | 4.188 | 4.348 | 2.465 | 3.103 | 5.311 | 5.414 | 7.451 | 8.485 |
| 3 | 4.889 | 4.635 | 2.671 | 2.584 | 4.565 | 5.027 | 7.264 | 8.157 |
| 3 | 3.959 | 4.629 | 1.726 | 2.131 | 5.445 | 3.625 | 6.901 | 8.795 |

^a^Mesial-distal length for crown area.

^b^Buccal-lingual length for crown area.

Data Table 2. Right P_3_s 2D ImageJ Dataset

| Lingual Cusp Number | ICD A-B (mm) | ICD A-C (mm) | ICD B-C (mm) | ICD C-D (mm) | ICD A-C (mm) | ICD B-D (mm) | MD^a^ (mm) | BL^b^ (mm) |
| --- | --- | --- | --- | --- | --- | --- | --- | --- |
| 1 | 4.914 | - | - | - | - | - | 7.045 | 8.839 |
| 1 | 4.298 | - | - | - | - | - | 7.162 | 7.926 |
| 1 | 3.521 | - | - | - | - | - | 6.813 | 7.582 |
| 1 | 3.913 | - | - | - | - | - | 6.828 | 8.563 |
| 1 | 4.547 | - | - | - | - | - | 7.76 | 8.920 |
| 1 | 3.358 | - | - | - | - | - | 7.313 | 8.759 |
| 1 | 3.878 | - | - | - | - | - | 7.108 | 8.511 |
| 1 | 3.871 | - | - | - | - | - | 6.983 | 8.549 |
| 1 | 4.616 | - | - | - | - | - | 6.37 | 8.091 |
| 1 | 3.824 | - | - | - | - | - | 7.149 | 9.563 |
| 1 | 4.110 | - | - | - | - | - | 5.993 | 7.789 |
| 1 | 3.765 | - | - | - | - | - | 5.968 | 7.870 |
| 1 | 3.849 | - | - | - | - | - | 7.117 | 9.150 |
| 1 | 4.454 | - | - | - | - | - | 7.450 | 8.256 |
| 1 | 3.681 | - | - | - | - | - | 7.756 | 8.052 |
| 1 | 5.201 | - | - | - | - | - | 8.114 | 9.807 |
| 1 | 3.722 | - | - | - | - | - | 7.076 | 8.907 |
| 1 | 3.809 | - | - | - | - | - | 7.106 | 7.366 |
| 1 | 4.567 | - | - | - | - | - | 7.050 | 8.096 |
| 1 | 4.336 | - | - | - | - | - | 6.887 | 9.072 |
| 1 | 3.530 | - | - | - | - | - | 7.397 | 8.569 |
| 1 | 3.790 | - | - | - | - | - | 7.639 | 7.826 |
| 1 | 3.125 | - | - | - | - | - | 6.590 | 8.156 |
| 1 | 4.026 | - | - | - | - | - | 7.055 | 8.324 |
| 1 | 3.424 | - | - | - | - | - | 7.192 | 8.726 |
| 1 | 3.213 | - | - | - | - | - | 7.091 | 7.962 |
| 1 | 3.539 | - | - | - | - | - | 6.409 | 7.830 |
| 1 | 3.299 | - | - | - | - | - | 6.410 | 7.469 |
| 1 | 5.032 | - | - | - | - | - | 7.163 | 8.254 |
| 1 | 4.396 | - | - | - | - | - | 7.729 | 9.796 |
| 2 | 4.377 | 4.243 | 2.787 | - | - | - | 7.484 | 8.547 |
| 2 | 5.597 | 4.357 | 4.777 | - | - | - | 7.206 | 8.748 |
| 2 | 4.245 | 5.369 | 2.849 | - | - | - | 6.353 | 8.554 |
| 2 | 4.091 | 5.492 | 3.081 | - | - | - | 7.770 | 8.554 |
| 2 | 4.070 | 4.705 | 3.897 | - | - | - | 7.748 | 9.125 |
| 2 | 4.399 | 5.197 | 3.685 | - | - | - | 6.596 | 8.764 |
| 2 | 4.025 | 4.928 | 3.644 | - | - | - | 6.834 | 8.162 |
| 2 | 3.651 | 4.787 | 2.797 | - | - | - | 7.286 | 8.542 |
| 2 | 4.180 | 6.131 | 3.862 | - | - | - | 7.139 | 9.361 |
| 2 | 4.612 | 5.059 | 3.521 | - | - | - | 6.823 | 8.820 |

Data Table 2 Continued.

| Lingual Cusp Number | ICD A-B (mm) | ICD A-C (mm) | ICD B-C (mm) | ICD C-D (mm) | ICD A-C (mm) | ICD B-D (mm) | MD^a^ (mm) | BL^b^ (mm) |
| --- | --- | --- | --- | --- | --- | --- | --- | --- |
| 2 | 4.033 | 5.235 | 3.375 | - | - | - | 6.638 | 7.930 |
| 2 | 3.645 | 4.179 | 3.809 | - | - | - | 6.983 | 8.783 |
| 2 | 4.634 | 5.401 | 3.110 | - | - | - | 7.630 | 9.271 |
| 2 | 4.921 | 5.585 | 3.564 | - | - | - | 7.542 | 9.194 |
| 2 | 3.816 | 5.404 | 3.732 | - | - | - | 7.878 | 9.568 |
| 2 | 3.785 | 4.319 | 3.065 | - | - | - | 7.283 | 8.848 |
| 2 | 4.511 | 5.242 | 3.739 | - | - | - | 6.760 | 9.243 |
| 2 | 4.128 | 4.773 | 2.148 | - | - | - | 6.635 | 8.698 |
| 2 | 4.158 | 4.920 | 2.975 | - | - | - | 7.361 | 9.288 |
| 2 | 4.030 | 4.324 | 3.757 | - | - | - | 7.010 | 8.041 |
| 2 | 4.410 | 5.525 | 3.623 | - | - | - | 7.362 | 8.717 |
| 2 | 5.131 | 4.876 | 1.427 | - | - | - | 6.519 | 8.123 |
| 2 | 3.607 | 4.551 | 3.303 | - | - | - | 7.271 | 8.533 |
| 2 | 4.143 | 4.553 | 2.751 | - | - | - | 7.330 | 7.398 |
| 2 | 4.101 | 5.198 | 2.380 | - | - | - | 7.631 | 8.770 |
| 2 | 3.488 | 3.936 | 2.901 | - | - | - | 6.337 | 7.192 |
| 2 | 3.210 | 3.820 | 2.601 | - | - | - | 6.334 | 7.241 |
| 2 | 3.367 | 4.169 | 2.828 | - | - | - | 6.804 | 8.304 |
| 2 | 2.000 | 3.111 | 2.612 | - | - | - | 7.788 | 8.111 |
| 2 | 3.347 | 4.219 | 2.617 | - | - | - | 5.990 | 7.424 |
| 2 | 3.410 | 3.402 | 2.709 | - | - | - | 6.136 | 8.012 |
| 2 | 4.141 | 5.183 | 2.018 | - | - | - | 6.914 | 8.852 |
| 3 | 4.370 | 4.172 | 2.547 | 2.475 | 4.558 | 5.005 | 7.921 | 8.385 |

^a^Mesial-distal length for crown area.

^b^Buccal-lingual length for crown area.

Data Table 3. Left P_4_s 2D ImageJ Dataset

| Lingual Cusp Number | ICD A-B (mm) | ICD A-C (mm) | ICD B-C (mm) | ICD C-D (mm) | ICD A-C (mm) | ICD B-D (mm) | MD^a^ (mm) | BL^b^ (mm) |
| --- | --- | --- | --- | --- | --- | --- | --- | --- |
| 1 | 5.356 | - | - | - | - | - | 6.644 | 8.401 |
| 1 | 4.894 | - | - | - | - | - | 7.801 | 9.262 |
| 1 | 4.146 | - | - | - | - | - | 7.489 | 8.765 |
| 1 | 4.790 | - | - | - | - | - | 7.181 | 9.800 |
| 1 | 4.351 | - | - | - | - | - | 6.772 | 9.065 |
| 1 | 4.298 | - | - | - | - | - | 6.713 | 8.991 |
| 1 | 4.460 | - | - | - | - | - | 6.543 | 7.142 |
| 1 | 4.506 | - | - | - | - | - | 7.601 | 8.982 |
| 1 | 4.383 | - | - | - | - | - | 7.061 | 8.584 |
| 1 | 4.605 | - | - | - | - | - | 7.717 | 8.834 |
| 2 | 4.762 | 5.669 | 3.833 | - | - | - | 6.877 | 9.201 |
| 2 | 5.552 | 6.120 | 4.893 | - | - | - | 7.898 | 9.000 |
| 2 | 4.585 | 5.359 | 4.274 | - | - | - | 7.463 | 9.342 |
| 2 | 3.890 | 4.685 | 4.873 | - | - | - | 7.498 | 9.492 |
| 2 | 5.200 | 4.976 | 4.007 | - | - | - | 7.229 | 9.053 |
| 2 | 4.976 | 5.297 | 5.192 | - | - | - | 7.199 | 9.404 |
| 2 | 5.652 | 4.624 | 4.195 | - | - | - | 7.162 | 8.308 |
| 2 | 4.323 | 6.079 | 4.908 | - | - | - | 8.545 | 9.828 |
| 2 | 4.529 | 5.588 | 5.243 | - | - | - | 7.437 | 9.451 |
| 2 | 4.809 | 5.446 | 4.133 | - | - | - | 7.140 | 8.573 |
| 2 | 4.782 | 4.441 | 5.030 | - | - | - | 6.574 | 8.733 |
| 2 | 5.176 | 5.632 | 4.334 | - | - | - | 7.356 | 9.163 |
| 2 | 5.257 | 5.497 | 4.510 | - | - | - | 7.741 | 10.37 |
| 2 | 6.089 | 5.541 | 5.356 | - | - | - | 7.612 | 10.15 |
| 2 | 4.725 | 5.456 | 5.714 | - | - | - | 7.896 | 9.930 |
| 2 | 3.989 | 4.710 | 4.500 | - | - | - | 7.825 | 10.12 |
| 2 | 3.935 | 4.712 | 3.905 | - | - | - | 7.351 | 9.684 |
| 2 | 4.194 | 5.319 | 4.372 | - | - | - | 7.170 | 9.191 |
| 2 | 3.947 | 4.067 | 2.719 | - | - | - | 8.038 | 8.501 |
| 2 | 4.564 | 4.866 | 3.778 | - | - | - | 7.137 | 8.046 |
| 2 | 4.799 | 5.120 | 4.733 | - | - | - | 7.610 | 9.533 |
| 2 | 3.773 | 4.653 | 2.921 | - | - | - | 7.217 | 8.934 |
| 2 | 4.291 | 4.700 | 4.292 | - | - | - | 7.112 | 8.958 |
| 2 | 5.207 | 5.707 | 4.359 | - | - | - | 7.746 | 8.712 |
| 2 | 3.755 | 4.433 | 3.536 | - | - | - | 8.151 | 8.309 |
| 2 | 3.684 | 3.760 | 2.273 | - | - | - | 7.863 | 8.000 |
| 2 | 4.079 | 3.662 | 4.018 | - | - | - | 6.838 | 7.630 |
| 2 | 4.201 | 4.944 | 3.608 | - | - | - | 6.198 | 8.457 |
| 2 | 4.391 | 4.665 | 3.581 | - | - | - | 7.147 | 8.456 |
| 2 | 4.898 | 5.328 | 3.678 | - | - | - | 8.171 | 7.062 |

Data Table 3 Continued.

| Lingual Cusp Number | ICD A-B (mm) | ICD A-C (mm) | ICD B-C (mm) | ICD C-D (mm) | ICD A-C (mm) | ICD B-D (mm) | MD^a^ (mm) | BL^b^ (mm) |
| --- | --- | --- | --- | --- | --- | --- | --- | --- |
| 2 | 5.047 | 5.181 | 4.850 | - | - | - | 6.616 | 8.060 |
| 2 | 5.354 | 5.674 | 4.721 | - | - | - | 7.498 | 8.903 |
| 2 | 4.245 | 4.486 | 3.399 | - | - | - | 7.861 | 9.350 |
| 2 | 4.218 | 4.864 | 4.749 | - | - | - | 7.371 | 8.162 |
| 2 | 4.197 | 3.852 | 3.958 | - | - | - | 7.018 | 8.734 |
| 2 | 4.755 | 4.722 | 3.522 | - | - | - | 6.847 | 7.924 |
| 2 | 5.848 | 5.229 | 3.769 | - | - | - | 6.845 | 8.803 |
| 3 | 5.133 | 4.918 | 3.151 | 2.671 | 4.501 | 5.583 | 7.901 | 9.614 |
| 3 | 4.777 | 5.213 | 2.053 | 2.723 | 4.789 | 4.530 | 7.043 | 8.743 |
| 3 | 5.108 | 6.022 | 3.348 | 2.462 | 5.622 | 5.282 | 7.296 | 9.541 |
| 3 | 2.815 | 4.730 | 3.381 | 5.183 | 5.235 | 6.922 | 8.015 | 8.678 |
| 3 | 5.032 | 4.989 | 2.748 | 2.845 | 5.040 | 5.362 | 7.171 | 9.828 |
| 3 | 5.093 | 5.568 | 2.734 | 2.567 | 5.037 | 4.759 | 7.488 | 9.759 |
| 3 | 5.159 | 5.402 | 3.442 | 2.180 | 4.794 | 4.984 | 8.481 | 9.916 |
| 3 | 4.335 | 4.794 | 3.064 | 2.389 | 4.826 | 5.260 | 7.243 | 9.006 |
| 3 | 3.927 | 5.178 | 3.620 | 2.417 | 4.835 | 5.161 | 7.556 | 9.287 |
| 3 | 3.575 | 4.323 | 3.171 | 2.388 | 3.908 | 4.855 | 6.789 | 9.800 |
| 3 | 4.550 | 5.490 | 3.225 | 1.977 | 5.035 | 4.578 | 7.518 | 8.712 |
| 3 | 4.558 | 5.255 | 3.281 | 1.914 | 4.806 | 4.75 | 7.036 | 8.447 |
| 3 | 4.074 | 4.904 | 2.670 | 1.763 | 4.161 | 3.756 | 6.989 | 8.665 |
| 3 | 4.602 | 4.937 | 2.798 | 2.130 | 4.551 | 4.292 | 7.412 | 7.247 |
| 3 | 4.431 | 4.496 | 2.153 | 2.211 | 3.959 | 4.012 | 6.634 | 7.548 |
| 3 | 4.461 | 5.697 | 3.648 | 2.975 | 4.730 | 5.550 | 7.607 | 8.735 |

^a^Mesial-distal length for crown area.

^b^Buccal-lingual length for crown area.

Data Table 4. Right P_4_s 2D ImageJ Dataset

| Lingual Cusp Number | ICD A-B (mm) | ICD A-C (mm) | ICD B-C (mm) | ICD C-D (mm) | ICD A-C (mm) | ICD B-D (mm) | MD^a^ (mm) | BL^b^ (mm) |
| --- | --- | --- | --- | --- | --- | --- | --- | --- |
| 1 | 5.449 | - | - | - | - | - | 7.004 | 9.224 |
| 1 | 4.559 | - | - | - | - | - | 7.253 | 8.601 |
| 1 | 4.450 | - | - | - | - | - | 7.348 | 8.417 |
| 1 | 4.543 | - | - | - | - | - | 7.313 | 8.759 |
| 1 | 4.037 | - | - | - | - | - | 7.059 | 8.896 |
| 1 | 3.641 | - | - | - | - | - | 7.033 | 9.137 |
| 1 | 4.476 | - | - | - | - | - | 7.508 | 7.945 |
| 1 | 4.934 | - | - | - | - | - | 7.338 | 7.534 |
| 1 | 3.547 | - | - | - | - | - | 7.458 | 9.223 |
| 2 | 5.704 | 6.347 | 5.000 | - | - | - | 7.454 | 9.602 |
| 2 | 5.541 | 5.537 | 3.351 | - | - | - | 7.014 | 8.466 |
| 2 | 4.743 | 6.354 | 4.946 | - | - | - | 8.102 | 8.636 |
| 2 | 4.367 | 5.389 | 5.039 | - | - | - | 7.167 | 9.596 |
| 2 | 5.261 | 4.967 | 3.672 | - | - | - | 7.612 | 9.376 |
| 2 | 5.288 | 5.420 | 6.205 | - | - | - | 7.350 | 9.789 |
| 2 | 5.128 | 4.595 | 4.032 | - | - | - | 7.458 | 8.555 |
| 2 | 5.324 | 5.399 | 5.514 | - | - | - | 7.668 | 9.467 |
| 2 | 4.974 | 5.457 | 4.375 | - | - | - | 7.450 | 8.900 |
| 2 | 4.242 | 4.477 | 4.597 | - | - | - | 6.876 | 8.726 |
| 2 | 5.309 | 5.044 | 4.564 | - | - | - | 7.330 | 8.747 |
| 2 | 4.712 | 4.671 | 4.762 | - | - | - | 7.828 | 9.975 |
| 2 | 3.904 | 5.340 | 4.601 | - | - | - | 7.530 | 10.11 |
| 2 | 5.809 | 5.641 | 5.263 | - | - | - | 7.270 | 10.07 |
| 2 | 5.231 | 5.845 | 4.896 | - | - | - | 7.469 | 9.960 |
| 2 | 4.208 | 4.523 | 4.653 | - | - | - | 6.744 | 9.32 |
| 2 | 3.686 | 4.731 | 4.400 | - | - | - | 6.571 | 9.238 |
| 2 | 4.747 | 4.909 | 4.886 | - | - | - | 6.955 | 8.645 |
| 2 | 5.289 | 5.800 | 5.363 | - | - | - | 7.334 | 9.692 |
| 2 | 4.749 | 5.207 | 3.318 | - | - | - | 6.982 | 9.759 |
| 2 | 4.436 | 4.563 | 3.281 | - | - | - | 7.523 | 9.337 |
| 2 | 4.973 | 5.155 | 5.032 | - | - | - | 7.432 | 9.022 |
| 2 | 4.497 | 5.087 | 4.766 | - | - | - | 6.967 | 9.172 |
| 2 | 3.920 | 4.813 | 3.283 | - | - | - | 6.425 | 8.859 |
| 2 | 4.359 | 5.031 | 4.819 | - | - | - | 8.182 | 8.902 |
| 2 | 5.547 | 5.738 | 4.857 | - | - | - | 7.724 | 8.177 |
| 2 | 3,778 | 4.492 | 2.254 | - | - | - | 7.264 | 7.105 |
| 2 | 4.711 | 4.459 | 4.461 | - | - | - | 8.197 | 8.319 |
| 2 | 4.106 | 3.811 | 3.830 | - | - | - | 6.486 | 8.485 |
| 2 | 4.979 | 5.469 | 3.037 | - | - | - | 7.884 | 9.132 |
| 2 | 4.650 | 4.651 | 3.024 | - | - | - | 6.942 | 8.074 |

Data Table 4 Continued.

| Lingual Cusp Number | ICD A-B (mm) | ICD A-C (mm) | ICD B-C (mm) | ICD C-D (mm) | ICD A-C (mm) | ICD B-D (mm) | MD^a^ (mm) | BL^b^ (mm) |
| --- | --- | --- | --- | --- | --- | --- | --- | --- |
| 2 | 3.824 | 4.560 | 3.549 | - | - | - | 7.425 | 9.329 |
| 2 | 4.944 | 5.062 | 2.983 | - | - | - | 7.063 | 7.973 |
| 2 | 4.616 | 5.522 | 5.194 | - | - | - | 7.631 | 8.318 |
| 2 | 3.831 | 4.588 | 4.646 | - | - | - | 6.354 | 8.737 |
| 2 | 4.156 | 5.113 | 3.557 | - | - | - | 7.438 | 8.053 |
| 2 | 4.188 | 5.135 | 2.302 | - | - | - | 7.764 | 8.224 |
| 2 | 4.317 | 4.508 | 2.706 | - | - | - | 6.856 | 7.634 |
| 2 | 4.408 | 4.109 | 4.040 | - | - | - | 6.768 | 7.722 |
| 2 | 4.821 | 4.346 | 4.399 | - | - | - | 6.758 | 8.627 |
| 2 | 5.702 | 4.929 | 4.030 | - | - | - | 7.205 | 10.13 |
| 3 | 5.046 | 5.314 | 3.158 | 3,277 | 6.111 | 6.271 | 7.419 | 9.349 |
| 3 | 5.084 | 6.405 | 2.788 | 2.441 | 5.932 | 4.467 | 7.553 | 9.695 |
| 3 | 4.339 | 4.932 | 4.693 | 3.731 | 2.769 | 5.749 | 8.296 | 8.759 |
| 3 | 5.443 | 5.576 | 2.765 | 2.104 | 4.779 | 4.601 | 8.036 | 9.517 |
| 3 | 3.298 | 3.845 | 2.652 | 2.483 | 4.025 | 4.930 | 6.680 | 8.238 |
| 3 | 3.640 | 4.801 | 2.784 | 3.436 | 4.535 | 5.203 | 7.751 | 10.19 |
| 3 | 4.540 | 5.036 | 3.265 | 2.275 | 4.501 | 4.843 | 7.064 | 9.285 |
| 3 | 4.530 | 5.175 | 3.174 | 1.975 | 4.465 | 4.675 | 7.620 | 9.752 |
| 3 | 3.126 | 4.292 | 2.760 | 2.702 | 4.005 | 4.738 | 6.998 | 7.437 |
| 3 | 4.750 | 4.740 | 1.827 | 1.841 | 5.539 | 3.843 | 7.001 | 9.339 |
| 3 | 4.088 | 4.439 | 2.679 | 1.832 | 3.657 | 3.896 | 6.514 | 8.869 |
| 3 | 3.530 | 4.364 | 2.530 | 3.495 | 4.725 | 5.262 | 7.462 | 7.324 |
| 3 | 4.795 | 5.606 | 4.472 | 2.776 | 4.525 | 5.657 | 7.709 | 9.129 |

^a^Mesial-distal length for crown area.

^b^Buccal-lingual length for crown area.

Data Table 5. Left P_3_s 3D MeshLab Dataset

| Lingual Cusp Number | ICD A-B (mm) | ICD A-C (mm) | ICD B-C (mm) | ICD C-D (mm) | ICD A-C (mm) | ICD B-D (mm) |
| --- | --- | --- | --- | --- | --- | --- |
| 1 | 3.767 | - | - | - | - | - |
| 1 | 4.614 | - | - | - | - | - |
| 1 | 4.158 | - | - | - | - | - |
| 1 | 3.487 | - | - | - | - | - |
| 1 | 3.997 | - | - | - | - | - |
| 1 | 3.888 | - | - | - | - | - |
| 1 | 3.915 | - | - | - | - | - |
| 1 | 3.493 | - | - | - | - | - |
| 1 | 3.794 | - | - | - | - | - |
| 1 | 4.041 | - | - | - | - | - |
| 1 | 4.996 | - | - | - | - | - |
| 1 | 5.584 | - | - | - | - | - |
| 1 | 4.370 | - | - | - | - | - |
| 1 | 3.860 | - | - | - | - | - |
| 1 | 4.771 | - | - | - | - | - |
| 1 | 4.939 | - | - | - | - | - |
| 1 | 4.304 | - | - | - | - | - |
| 1 | 3.451 | - | - | - | - | - |
| 1 | 4.610 | - | - | - | - | - |
| 1 | 3.493 | - | - | - | - | - |
| 1 | 4.902 | - | - | - | - | - |
| 1 | 3.462 | - | - | - | - | - |
| 1 | 3.402 | - | - | - | - | - |
| 1 | 5.564 | - | - | - | - | - |
| 1 | 5.461 | - | - | - | - | - |
| 2 | 5.055 | 5.799 | 3.751 | - | - | - |
| 2 | 4.525 | 5.033 | 2.706 | - | - | - |
| 2 | 5.348 | 5.466 | 2.797 | - | - | - |
| 2 | 4.760 | 4.438 | 2.524 | - | - | - |
| 2 | 4.255 | 5.698 | 3.790 | - | - | - |
| 2 | 4.038 | 6.028 | 3.493 | - | - | - |
| 2 | 3.666 | 5.630 | 4.313 | - | - | - |
| 2 | 5.337 | 5.524 | 2.635 | - | - | - |
| 2 | 3.987 | 4.806 | 2.782 | - | - | - |
| 2 | 5.537 | 5.857 | 2.542 | - | - | - |
| 2 | 4.816 | 4.990 | 3.236 | - | - | - |
| 2 | 5.249 | 6.202 | 3.756 | - | - | - |
| 2 | 4.668 | 6.164 | 4.532 | - | - | - |
| 2 | 3.960 | 5.474 | 2.727 | - | - | - |
| 2 | 4.946 | 5.826 | 3.924 | - | - | - |
| 2 | 5.419 | 5.523 | 2.148 | - | - | - |

Data Table 5 Continued.

| Lingual Cusp Number | ICD A-B (mm) | ICD A-C (mm) | ICD B-C (mm) | ICD C-D (mm) | ICD A-C (mm) | ICD B-D (mm) |
| --- | --- | --- | --- | --- | --- | --- |
| 2 | 4.047 | 4.835 | 3.212 | - | - | - |
| 2 | 3.951 | 5.492 | 3.570 | - | - | - |
| 2 | 4.926 | 4.903 | 2.067 | - | - | - |
| 2 | 5.069 | 5.285 | 2.692 | - | - | - |
| 2 | 4.492 | 5.605 | 2.751 | - | - | - |
| 2 | 3.828 | 4.654 | 2.268 | - | - | - |
| 2 | 3.471 | 4.691 | 3.036 | - | - | - |
| 2 | 3.900 | 4.581 | 1.968 | - | - | - |
| 2 | 4.367 | 4.029 | 3.103 | - | - | - |
| 2 | 3.880 | 3.788 | 2.839 | - | - | - |
| 2 | 4.919 | 5.216 | 2.231 | - | - | - |
| 2 | 4.229 | 4.990 | 2.300 | - | - | - |
| 2 | 3.706 | 4.913 | 3.370 | - | - | - |
| 2 | 4.657 | 4.804 | 2.860 | - | - | - |
| 2 | 4.592 | 5.088 | 2.783 | - | - | - |
| 2 | 4.04 | 4.754 | 2.821 | - | - | - |
| 2 | 3.552 | 4.262 | 3.217 | - | - | - |
| 2 | 3.617 | 5.614 | 2.565 | - | - | - |
| 2 | 4.008 | 4.721 | 3.463 | - | - | - |
| 3 | 5.613 | 4.903 | 2.980 | 2.224 | 5.456 | 5.239 |
| 3 | 4.231 | 4.466 | 2.758 | 2.380 | 4.861 | 4.634 |
| 3 | 4.275 | 5.070 | 1.509 | 1.718 | 5.496 | 3.255 |

Data Table 6. Right P_3_s 3D MeshLab Dataset

| Lingual Cusp Number | ICD A-B (mm) | ICD A-C (mm) | ICD B-C (mm) | ICD C-D (mm) | ICD A-C (mm) | | ICD B-D (mm) |
| --- | --- | --- | --- | --- | --- | --- | --- |
| 1 | 4.910 | - | - | - | - | - | |
| 1 | 4.142 | - | - | - | - | - | |
| 1 | 3.965 | - | - | - | - | - | |
| 1 | 4.266 | - | - | - | - | - | |
| 1 | 5.424 | - | - | - | - | - | |
| 1 | 3.848 | - | - | - | - | - | |
| 1 | 3.773 | - | - | - | - | - | |
| 1 | 4.008 | - | - | - | - | - | |
| 1 | 5.290 | - | - | - | - | - | |
| 1 | 3.981 | - | - | - | - | - | |
| 1 | 3.656 | - | - | - | - | - | |
| 1 | 4.034 | - | - | - | - | - | |
| 1 | 4.081 | - | - | - | - | - | |
| 1 | 4.915 | - | - | - | - | - | |
| 1 | 4.536 | - | - | - | - | - | |
| 1 | 5.298 | - | - | - | - | - | |
| 1 | 4.114 | - | - | - | - | - | |
| 1 | 3.731 | - | - | - | - | - | |
| 1 | 4.119 | - | - | - | - | - | |
| 1 | 4.829 | - | - | - | - | - | |
| 1 | 4.161 | - | - | - | - | - | |
| 1 | 4.490 | - | - | - | - | - | |
| 1 | 4.224 | - | - | - | - | - | |
| 1 | 5.089 | - | - | - | - | - | |
| 1 | 4.926 | - | - | - | - | - | |
| 1 | 3.759 | - | - | - | - | - | |
| 1 | 3.753 | - | - | - | - | - | |
| 1 | 3.366 | - | - | - | - | - | |
| 1 | 3.648 | - | - | - | - | - | |
| 1 | 5.594 | - | - | - | - | - | |
| 1 | 5.091 | - | - | - | - | - | |
| 2 | 4.304 | 5.366 | 3.503 | - | - | - | |
| 2 | 4.337 | 5.086 | 2.080 | - | - | - | |
| 2 | 4.936 | 4.483 | 4.793 | - | - | - | |
| 2 | 4.407 | 5.549 | 3.112 | - | - | - | |
| 2 | 3.668 | 5.280 | 3.675 | - | - | - | |

Data Table 6 Continued.

| Lingual Cusp Number | ICD A-B (mm) | ICD A-C (mm) | ICD B-C (mm) | ICD C-D (mm) | ICD A-C (mm) | ICD B-D (mm) |
| --- | --- | --- | --- | --- | --- | --- |
| 2 | 4.812 | 4.855 | 2.482 | - | - | - |
| 2 | 3.906 | 5.229 | 3.207 | - | - | - |
| 2 | 4.534 | 5.692 | 3.388 | - | - | - |
| 2 | 3.994 | 5.544 | 3.014 | - | - | - |
| 2 | 4.234 | 6.399 | 3.964 | - | - | - |
| 2 | 4.842 | 5.624 | 3.528 | - | - | - |
| 2 | 3.462 | 4.581 | 3.948 | - | - | - |
| 2 | 5.205 | 5.931 | 3.582 | - | - | - |
| 2 | 4.768 | 6.153 | 3.873 | - | - | - |
| 2 | 4.005 | 5.652 | 3.751 | - | - | - |
| 2 | 3.602 | 3.985 | 3.102 | - | - | - |
| 2 | 4.987 | 6.157 | 3.920 | - | - | - |
| 2 | 4.650 | 5.489 | 2.281 | - | - | - |
| 2 | 4.314 | 5.136 | 3.195 | - | - | - |
| 2 | 3.486 | 4.569 | 3.076 | - | - | - |
| 2 | 4.390 | 4.894 | 2.745 | - | - | - |
| 2 | 3.889 | 4.000 | 2.698 | - | - | - |
| 2 | 3.695 | 4.855 | 4.106 | - | - | - |
| 2 | 4.372 | 5.384 | 3.139 | - | - | - |
| 2 | 5.249 | 5.177 | 1.918 | - | - | - |
| 2 | 4.006 | 5.033 | 3.481 | - | - | - |
| 2 | 4.336 | 4.894 | 3.089 | - | - | - |
| 2 | 4.973 | 5.858 | 2.421 | - | - | - |
| 2 | 3.727 | 4.521 | 2.576 | - | - | - |
| 2 | 3.665 | 4.445 | 2.691 | - | - | - |
| 2 | 4.478 | 5.369 | 2.030 | - | - | - |
| 3 | 5.348 | 4.791 | 2.394 | 2.544 | 5.373 | 4.871 |

Data Table 7. Left P_4_s 3D MeshLab Dataset.

| Lingual Cusp Number | ICD A-B (mm) | ICD A-C (mm) | ICD B-C (mm) | ICD C-D (mm) | ICD A-C (mm) | ICD B-D (mm) |
| --- | --- | --- | --- | --- | --- | --- |
| 1 | 5.022 | - | - | - | - | - |
| 1 | 4.208 | - | - | - | - | - |
| 1 | 4.759 | - | - | - | - | - |
| 1 | 4.234 | - | - | - | - | - |
| 1 | 4.412 | - | - | - | - | - |
| 1 | 4.531 | - | - | - | - | - |
| 1 | 4.467 | - | - | - | - | - |
| 1 | 4.630 | - | - | - | - | - |
| 1 | 4.808 | - | - | - | - | - |
| 1 | 4.712 | - | - | - | - | - |
| 2 | 4.886 | 5.879 | 4.178 | - | - | - |
| 2 | 5.017 | 5.639 | 3.809 | - | - | - |
| 2 | 4.425 | 4.853 | 4.142 | - | - | - |
| 2 | 4.276 | 5.284 | 4.805 | - | - | - |
| 2 | 4.291 | 5.646 | 4.428 | - | - | - |
| 2 | 4.211 | 4.313 | 3.078 | - | - | - |
| 2 | 5.132 | 4.899 | 3.855 | - | - | - |
| 2 | 5.014 | 5.696 | 4.738 | - | - | - |
| 2 | 3.704 | 4.885 | 3.948 | - | - | - |
| 2 | 4.806 | 5.365 | 3.952 | - | - | - |
| 2 | 3.908 | 4.501 | 3.673 | - | - | - |
| 2 | 5.132 | 5.888 | 4.232 | - | - | - |
| 2 | 5.332 | 5.349 | 3.100 | - | - | - |
| 2 | 5.213 | 5.836 | 4.336 | - | - | - |
| 2 | 4.013 | 4.976 | 4.725 | - | - | - |
| 2 | 4.491 | 5.177 | 3.412 | - | - | - |
| 2 | 5.717 | 6.157 | 4.271 | - | - | - |
| 2 | 5.014 | 5.588 | 4.282 | - | - | - |
| 2 | 4.539 | 6.031 | 4.382 | - | - | - |
| 2 | 4.023 | 5.897 | 5.117 | - | - | - |
| 2 | 3.949 | 5.036 | 4.735 | - | - | - |
| 2 | 4.860 | 4.822 | 4.369 | - | - | - |
| 2 | 5.851 | 5.337 | 4.803 | - | - | - |
| 2 | 4.448 | 6.435 | 4.894 | - | - | - |
| 2 | 4.546 | 6.458 | 4.136 | - | - | - |
| 2 | 4.894 | 5.503 | 5.348 | - | - | - |

Data Table 7 Continued.

| Lingual Cusp Number | ICD A-B (mm) | ICD A-C (mm) | ICD B-C (mm) | ICD C-D (mm) | ICD A-C (mm) | ICD B-D (mm) |
| --- | --- | --- | --- | --- | --- | --- |
| 2 | 5.099 | 5.705 | 4.373 | - | - | - |
| 2 | 6.552 | 6.768 | 5.286 | - | - | - |
| 2 | 4.369 | 5.253 | 4.990 | - | - | - |
| 2 | 3.992 | 5.239 | 4.240 | - | - | - |
| 2 | 3.754 | 5.044 | 3.215 | - | - | - |
| 2 | 3.878 | 4.292 | 2.614 | - | - | - |
| 2 | 3.668 | 3.738 | 3.191 | - | - | - |
| 2 | 4.149 | 4.179 | 2.727 | - | - | - |
| 2 | 5.544 | 6.236 | 3.675 | - | - | - |
| 2 | 4.720 | 5.274 | 4.681 | - | - | - |
| 2 | 3.999 | 4.506 | 4.623 | - | - | - |
| 2 | 5.517 | 5.393 | 4.170 | - | - | - |
| 3 | 5.549 | 5.554 | 3.344 | 2.576 | 4.661 | 5.274 |
| 3 | 4.628 | 5.616 | 2.519 | 2.657 | 5.334 | 4.466 |
| 3 | 5.152 | 5.799 | 3.550 | 2.171 | 5.429 | 5.300 |
| 3 | 3.860 | 4.451 | 3.298 | 2.268 | 5.138 | 4.595 |
| 3 | 4.205 | 5.424 | 3.100 | 1.955 | 5.119 | 4.343 |
| 3 | 4.294 | 5.236 | 2.850 | 2.093 | 4.385 | 4.037 |
| 3 | 4.274 | 4.825 | 2.227 | 2.275 | 4.259 | 4.100 |
| 3 | 4.704 | 5.349 | 1.749 | 2.501 | 4.702 | 4.229 |
| 3 | 4.916 | 5.685 | 3.426 | 2.182 | 5.179 | 4.916 |
| 3 | 4.575 | 5.537 | 2.041 | 2.878 | 5.662 | 4.436 |
| 3 | 4.183 | 5.922 | 3.701 | 2.397 | 4.720 | 5.429 |
| 3 | 3.858 | 5.568 | 3.339 | 2.266 | 5.360 | 4.800 |
| 3 | 4.722 | 5.298 | 2.738 | 1.883 | 5.027 | 4.288 |
| 3 | 4.571 | 4.852 | 2.534 | 2.119 | 4.662 | 4.329 |
| 3 | 4.763 | 6.028 | 3.724 | 2.957 | 5.145 | 5.511 |

Data Table 8. Right P_4_s 3D MeshLab Dataset

| Lingual Cusp Number | ICD A-B (mm) | ICD A-C (mm) | ICD B-C (mm) | ICD C-D (mm) | ICD A-C (mm) | ICD B-D (mm) |
| --- | --- | --- | --- | --- | --- | --- |
| 1 | 4.758 | - | - | - | - | - |
| 1 | 4.316 | - | - | - | - | - |
| 1 | 4.691 | - | - | - | - | - |
| 1 | 4.176 | - | - | - | - | - |
| 1 | 4.061 | - | - | - | - | - |
| 1 | 5.160 | - | - | - | - | - |
| 1 | 5.071 | - | - | - | - | - |
| 1 | 4.734 | - | - | - | - | - |
| 1 | 4.397 | - | - | - | - | - |
| 2 | 4.634 | 5.103 | 2.618 | - | - | - |
| 2 | 4.041 | 4.449 | 2.295 | - | - | - |
| 2 | 5.102 | 5.310 | 2.981 | - | - | - |
| 2 | 4.485 | 4.288 | 3.184 | - | - | - |
| 2 | 4.387 | 5.089 | 2.471 | - | - | - |
| 2 | 4.782 | 5.578 | 3.817 | - | - | - |
| 2 | 4.171 | 4.252 | 4.644 | - | - | - |
| 2 | 4.411 | 4.902 | 4.841 | - | - | - |
| 2 | 4.531 | 5.272 | 4.219 | - | - | - |
| 2 | 4.484 | 4.928 | 3.253 | - | - | - |
| 2 | 4.752 | 5.380 | 4.323 | - | - | - |
| 2 | 4.164 | 5.549 | 5.118 | - | - | - |
| 2 | 5.482 | 6.083 | 4.771 | - | - | - |
| 2 | 4.770 | 4.742 | 4.469 | - | - | - |
| 2 | 4.739 | 5.017 | 3.294 | - | - | - |
| 2 | 4.304 | 5.507 | 4.749 | - | - | - |
| 2 | 3.761 | 4.808 | 4.634 | - | - | - |
| 2 | 3.934 | 4.611 | 4.064 | - | - | - |
| 2 | 5.664 | 5.615 | 3.962 | - | - | - |
| 2 | 4.985 | 5.114 | 4.583 | - | - | - |
| 2 | 5.744 | 5.537 | 5.351 | - | - | - |
| 2 | 4.911 | 6.162 | 5.079 | - | - | - |
| 2 | 4.877 | 4.361 | 4.703 | - | - | - |
| 2 | 5.004 | 5.847 | 5.910 | - | - | - |
| 2 | 5.250 | 4.795 | 4.093 | - | - | - |
| 2 | 4.978 | 5.560 | 4.581 | - | - | - |
| 2 | 4.230 | 5.322 | 4.437 | - | - | - |

Data Table 8 Continued.

| Lingual Cusp Number | ICD A-B (mm) | ICD A-C (mm) | ICD B-C (mm) | ICD C-D (mm) | ICD A-C (mm) | ICD B-D (mm) |
| --- | --- | --- | --- | --- | --- | --- |
| 2 | 6.649 | 6.230 | 5.575 | - | - | - |
| 2 | 4.806 | 5.853 | 4.885 | - | - | - |
| 2 | 3.394 | 4.553 | 3.740 | - | - | - |
| 2 | 3.939 | 5.054 | 3.331 | - | - | - |
| 2 | 4.485 | 4.139 | 3.946 | - | - | - |
| 2 | 4.611 | 5.267 | 3.118 | - | - | - |
| 2 | 3.965 | 5.041 | 3.816 | - | - | - |
| 2 | 4.530 | 4.036 | 4.373 | - | - | - |
| 2 | 5.829 | 5.428 | 3.890 | - | - | - |
| 2 | 4.234 | 5.099 | 3.804 | - | - | - |
| 2 | 4.620 | 5.552 | 4.970 | - | - | - |
| 2 | 4.191 | 4.719 | 2.821 | - | - | - |
| 2 | 5.205 | 6.269 | 4.847 | - | - | - |
| 2 | 5.021 | 4.822 | 3.163 | - | - | - |
| 3 | 3.720 | 3.876 | 2.255 | 2.881 | 4.279 | 4.651 |
| 3 | 4.653 | 5.297 | 2.250 | 1.538 | 5.744 | 3.697 |
| 3 | 5.080 | 4.319 | 2.746 | 3.523 | 5.926 | 6.062 |
| 3 | 4.871 | 5.424 | 3.394 | 1.197 | 4.959 | 5.306 |
| 3 | 3.579 | 4.235 | 2.393 | 2.751 | 4.239 | 4.504 |
| 3 | 6.211 | 6.534 | 3.083 | 2.344 | 5.324 | 4.736 |
| 3 | 4.228 | 5.589 | 3.326 | 3.192 | 5.333 | 5.407 |
| 3 | 4.421 | 5.127 | 2.683 | 1.990 | 4.280 | 3.754 |
| 3 | 5.325 | 6.642 | 3.084 | 2.463 | 5.584 | 4.672 |
| 3 | 4.643 | 5.097 | 3.557 | 2.068 | 4.738 | 4.983 |
| 3 | 4.719 | 5.258 | 3.547 | 1.888 | 4.556 | 4.819 |
| 3 | 3.825 | 4.473 | 2.373 | 3.436 | 4.780 | 5.221 |
| 3 | 4.750 | 5.567 | 3.841 | 2.397 | 4.912 | 5.321 |

Data Table 9. Cusp Area for Two Lingual Cusped P_4_s

| Cusp A (mm^2^) | Cusp B (mm^2^) | Cusp C (mm^2^) |
| --- | --- | --- |
| 31.073 | 15.328 | 6.916 |
| 34.201 | 15.612 | 6.758 |
| 30.610 | 15.950 | 8.764 |
| 30.109 | 16.806 | 6.736 |
| 31.149 | 16.024 | 6.690 |
| 28.607 | 15.380 | 11.431 |
| 26.618 | 11.948 | 3.964 |
| 31.566 | 19.524 | 11.436 |
| 31.449 | 14.794 | 8.430 |
| 26.490 | 13.753 | 5.861 |
| 25.458 | 11.469 | 8.930 |
| 28.415 | 13.580 | 8.736 |
| 32.513 | 17.286 | 11.248 |
| 33.796 | 18.407 | 13.719 |
| 31.355 | 14.457 | 11.423 |
| 31.860 | 14.205 | 9.532 |
| 28.394 | 15.891 | 10.412 |
| 25.399 | 17.879 | 10.505 |
| 31.895 | 10.274 | 3.685 |
| 33.681 | 12.342 | 15.194 |
| 30.582 | 14.517 | 8.371 |
| 29.676 | 14.808 | 5.750 |
| 30.764 | 16.161 | 7.657 |
| 29.928 | 16.156 | 5.273 |
| 31.136 | 15.504 | 5.874 |
| 25.125 | 6.356 | 5.196 |
| 24.561 | 13.799 | 4.610 |
| 29.716 | 14.019 | 6.802 |
| 27.224 | 9.943 | 6.654 |
| 37.512 | 12.844 | 7.250 |
| 26.298 | 10.921 | 4.998 |
| 31.111 | 14.926 | 8.341 |
| 27.944 | 12.287 | 9.139 |
| 27.728 | 13.647 | 9.923 |
| 24.559 | 12.641 | 4.216 |
| 30.414 | 12.421 | 6.562 |

Data Table 9 Continued.

| Cusp A (mm^2^) | Cusp B (mm^2^) | Cusp C (mm^2^) |
| --- | --- | --- |
| 36.121 | 15.017 | 5.471 |
| 31.495 | 17.027 | 11.305 |
| 27.227 | 12.362 | 5.958 |
| 30.007 | 15.815 | 6.411 |
| 31.358 | 16.365 | 6.359 |
| 33.350 | 12.148 | 11.187 |
| 30.587 | 14.935 | 11.653 |
| 25.900 | 13.617 | 6.049 |
| 30.366 | 19.606 | 10.063 |
| 28.456 | 14.474 | 8.947 |
| 24.894 | 11.644 | 9.257 |
| 29.785 | 12.905 | 9.903 |
| 31.307 | 15.811 | 5.483 |
| 33.759 | 18.467 | 10.282 |
| 32.753 | 17.325 | 12.208 |
| 32.061 | 18.911 | 11.616 |
| 32.076 | 14.326 | 7.448 |
| 27.658 | 14.958 | 8.436 |
| 29.473 | 11.638 | 8.756 |
| 32.628 | 14.389 | 14.123 |
| 31.695 | 12.195 | 12.950 |
| 34.523 | 12.868 | 11.147 |
| 31.597 | 15.444 | 8.433 |
| 32.096 | 13.231 | 10.161 |
| 29.150 | 14.695 | 5.600 |
| 30.764 | 15.641 | 8.038 |
| 27.653 | 15.743 | 8.138 |
| 25.881 | 4.439 | 6.538 |
| 32.122 | 15.353 | 6.604 |
| 24.167 | 14.063 | 4.360 |
| 35.255 | 14.391 | 6.115 |
| 26.381 | 11.248 | 5.405 |
| 21.057 | 8.519 | 10.128 |
| 31.257 | 16.701 | 9.864 |
| 25.903 | 12.317 | 10.806 |
| 24.043 | 14.083 | 8.194 |

Data Table 9 Continued.

| Cusp A (mm^2^) | Cusp B (mm^2^) | Cusp C (mm^2^) |
| --- | --- | --- |
| 30.107 | 3.717 | 14.163 |
| 29.460 | 7.894 | 8.312 |
| 23.104 | 7.303 | 8.382 |
| 24.699 | 12.974 | 4.170 |
| 27.396 | 11.460 | 4.513 |
| 36.784 | 15.896 | 7.213 |

Data Table 10. Cusp Area for Three Lingual Cusped P_4_s

| Cusp A (mm^2^) | Cusp B (mm^2^) | Cusp C (mm^2^) | Cusp D (mm^2^) |
| --- | --- | --- | --- |
| 32.522 | 8.213 | 4.677 | 4.068 |
| 34.967 | 10.495 | 4.151 | 8.686 |
| 28.754 | 2.834 | 12.652 | 9.423 |
| 40.266 | 10.161 | 6.146 | 5.511 |
| 35.345 | 9.120 | 6.091 | 10.001 |
| 34.930 | 10.409 | 7.629 | 6.048 |
| 38.539 | 13.556 | 9.527 | 4.579 |
| 31.558 | 10.723 | 6.099 | 5.721 |
| 36.381 | 10.913 | 6.132 | 3.561 |
| 35.258 | 11.712 | 4.189 | 8.150 |
| 32.191 | 9.820 | 4.416 | 3.353 |
| 23.64 | 8.722 | 4.459 | 5.128 |
| 27.677 | 7.800 | 5.255 | 5.360 |
| 24.934 | 5.574 | 3.919 | 5.045 |
| 23.145 | 7.529 | 4.800 | 3.788 |
| 27.180 | 10.443 | 6.655 | 6.355 |
| 29.987 | 4.003 | 10.653 | 9.149 |
| 33.595 | 12.457 | 4.464 | 9.284 |
| 27.564 | 2.137 | 12.773 | 8.509 |
| 39.386 | 10.197 | 6.039 | 4.915 |
| 29.100 | 3.167 | 7.866 | 4.773 |
| 40.110 | 13.352 | 8.164 | 4.699 |
| 35.909 | 11.082 | 4.093 | 4.095 |
| 40.097 | 10.100 | 4.245 | 5.513 |
| 25.715 | 3.315 | 6.603 | 3.567 |
| 29.802 | 8.692 | 5.190 | 11.073 |
| 30.577 | 8.677 | 3.450 | 4.499 |
| 23.256 | 7.352 | 6.843 | 2.532 |
| 29.214 | 13.259 | 7.342 | 3.557 |
