# Supplementary material for: The Patterning Cascade Model and Human Mandibular Premolar Variation
Source: Am J Biol Anthropol. 2025 Dec 9;188(4):e70178. doi: 10.1002/ajpa.70178 (PMC12687927; doi:10.1002/ajpa.70178)
Supplement: Supplementary file 2 — Table S1: Results of the paired t‐tests from the intra‐observer error analysis. Table S2: Software used for each statistical analysis. Table S3: Descriptive statistics for P3 and P4 variables by lingual cusp number. Table S4: Descriptive statistics for lingual cusp number in P3 and P4. Table S5: Results of the antimeric analyses for left and right elements. Table S6: Independent t‐test results comparing mean A‐B‐C cusp angle in two and three lingual cusped teeth (bootstrap resampling applied). Figure S1: Occlusal views of a P4 crown with topographic overlay in MeshLab (I) and ImageJ (II). (I) Cusp tip locations are approximated and marked by black dots with reference to the curvature map and regional maxima along the z‐axis with adjustments made for wear or alternative cusp morphology as viewed from the original images. Solid black lines between cusp tips represent intercusp distances. (II) Cusp areas traced using ImageJ's freehand selection/trace tool, with shading used to distinguish the buccal (blue), mesiolingual (yellow), and distolingual (red) cusps. Figure S2: Bar graph illustrating raw measurements from the intra‐observer error study for ICDs in MeshLab. Figure S3: Bar graphs illustrating raw measurements from the intra‐observer error study for crown area in MeshLab. Top (A) is mesial‐distal length, and bottom (B) is buccal‐lingual length. Figure S4: Bar graph illustrating raw measurements from the intra‐observer error study for ICDs in ImageJ. Figure S5: Bar graphs illustrating raw measurements from the intra‐observer error study for crown area in ImageJ. Top (A) is mesial‐distal length, and bottom (B) is buccal‐lingual length. Figure S6: Pie charts comparing (A) P3 sample by lingual cusp number, and (B) P4 sample by lingual cusp number. Figure S7: Crown area by lingual cusp number for (A) P3s, and (B) P4s. Figure S8: Pie charts comparing (A) two lingual cusped P4 by average RCA, and (B) three lingual cusped P4 by average RCA. [file AJPA-188-e70178-s001.docx]

# SUPPLEMENTAL TABLES

Table S1. Results of the paired t-tests from the intra-observer error analysis

| Measurement | *p*-value^a^ | *t*-statistic | df | Error (%) |
| --- | --- | --- | --- | --- |
| MeshLab ICD | 0.70 | 0.38 | 57 | 2.02 |
| MeshLab Crown Area  (Buccal-Lingual) | 0.51 | 0.67 | 20 | 2.98 |
| MeshLab Crown Area  (Mesial-Distal) | **<0.01** | **2.09** | **20** | **1.06** |
| ImageJ ICD | 0.90 | 0.12 | 57 | 1.05 |
| ImageJ Crown Area  (Buccal-Lingual) | 0.86 | 0.43 | 20 | 0.23 |
| ImageJ Crown Area  (Mesial-Distal) | 0.90 | 0.45 | 20 | 0.36 |

^a^Statistically significant results (*p*<0.05) are bolded.

Table S2. Software used for each statistical analysis

| Statistcal Analysis | Software Used |
| --- | --- |
| Determination of normality | SPSS |
| F-Tests of equality of variance | Excel |
| Independent t-tests | SPSS |
| Mann-Whitney U-test | SPSS |
| Paired t-tests | SPSS |
| Wilcoxon signed ranks test | SPSS |
| ANOVA | SPSS |
| GLM regression | R Studio |
| Linear regression | R Studio |

#

Table S3. Descriptive statistics for P_3_ and P_4_ variables by lingual cusp number

| Lingual Cusp Number | Measurement^a^ | Mean | Standard Deviation | Variance |
| --- | --- | --- | --- | --- |
| P_3_ 3D Data | | | | |
| 1 | RICD A-B | 0.562 | 0.077 | 0.006 |
| 2 | RICD A-B | 0.562 | 0.069 | 0.005 |
|  | RICD A-C | 0.667 | 0.063 | 0.004 |
|  | RICD B-C | 0.396 | 0.080 | 0.006 |
| 3 | RICD A-B | 0.615 | 0.080 | 0.006 |
|  | RICD A-C | 0.609 | 0.032 | 0.001 |
|  | RICD B-C | 0.305 | 0.082 | 0.007 |
|  | RICD A-D | 0.671 | 0.043 | 0.002 |
|  | RICD B-D | 0.569 | 0.032 | 0.001 |
|  | RICD C-D | 0.280 | 0.998 | 0.011 |
| P_3_ 2D Data | | | | |
| 1 | Crown Area | 59.311 | 7.559 | 57.138 |
|  | RICD A-B | 0.518 | 0.068 | 0.005 |
| 2 | Crown Area | 60.530 | 7.881 | 66.110 |
|  | RICD A-B | 0.522 | 0.073 | 0.005 |
|  | RICD A-C | 0.605 | 0.074 | 0.006 |
|  | RICD B-C | 0.394 | 0.075 | 0.006 |
|  | A-B-C Cusp Angle | 33.844 | 5.896 | 34.767 |
| 3 | Crown Area | 62.397 | 2.722 | 7.409 |
|  | RICD A-B | 0.551 | 0.056 | 0.003 |
|  | RICD A-C | 0.564 | 0.042 | 0.002 |
|  | RICD B-C | 0.298 | 0.054 | 0.003 |
|  | RICD A-D | 0.630 | 0.050 | 0.003 |
|  | RICD B-D | 0.603 | 0.065 | 0.004 |
|  | RICD C-D | 0.326 | 0.096 | 0.009 |
|  | A-B-C Cusp Angle | 25.150 | 4.414 | 19.489 |
| P_4_ 3D Data | | | | |
| 1 | RICD A-B | 0.581 | 0.051 | 0.003 |
| 2 | RICD A-B | 0.579 | 0.074 | 0.006 |
|  | RICD A-C | 0.649 | 0.061 | 0.004 |
|  | RICD B-C | 0.512 | 0.087 | 0.008 |
| 3 | RICD A-B | 0.563 | 0.055 | 0.003 |
|  | RICD A-C | 0.651 | 0.060 | 0.004 |
|  | RICD B-C | 0.362 | 0.060 | 0.004 |
|  | RICD A-D | 0.612 | 0.069 | 0.005 |
|  | RICD B-D | 0.585 | 0.047 | 0.002 |
|  | RICD C-D | 0.295 | 0.061 | 0.004 |
| P_4_ 2D Data | | | | |
| 1 | Crown Area | 62.804 | 5.820 | 33.872 |
|  | RICD A-B | 0.570 | 0.071 | 0.005 |
| 2 | Crown Area | 65.585 | 7.357 | 54.125 |
|  | RICD A-B | 0.580 | 0.067 | 0.005 |
|  | RICD A-C | 0.623 | 0.057 | 0.003 |
|  | RICD B-C | 0.520 | 0.093 | 0.009 |
|  | Cusp A Area | 0.455 | 0.035 | 0.001 |
|  | Cusp B Area | 0.212 | 0.038 | 0.001 |
|  | Cusp C Area | 0.126 | 0.036 | 0.001 |
|  | A-B-C Cusp Angle | 42.659 | 6.151 | 37.835 |
| 3 | Crown Area | 66.141 | 8.546 | 73.034 |
|  | RICD A-B | 0.539 | 0.074 | 0.006 |
|  | RICD A-C | 0.617 | 0.057 | 0.003 |
|  | RICD B-C | 0.371 | 0.068 | 0.005 |
|  | RICD A-D | 0.572 | 0.086 | 0.007 |
|  | RICD B-D | 0.601 | 0.080 | 0.006 |
|  | RICD C-D | 0.308 | 0.080 | 0.006 |
|  | Cusp A Area | 0.477 | 0.047 | 0.002 |
|  | Cusp B Area | 0.131 | 0.042 | 0.002 |
|  | Cusp C Area | 0.096 | 0.034 | 0.001 |
|  | Cusp D Area | 0.088 | 0.032 | 0.001 |
|  | A-B-C Cusp Angle | 27.728 | 4.701 | 22.095 |

^a^Dimensional values are in millimeters (mm) with the exception of areas (mm^2^) and angles (degrees).

# Table S4. Descriptive statistics for lingual cusp number in P_3_ and P_4_

| Tooth | One Lingual Cusp | Two Lingual Cusps | Three Lingual Cusps | Total Sample |
| --- | --- | --- | --- | --- |
| Left P_3_ | 26 (41%) | 34 (54%) | 3 (5%) | 63 |
| Right P_3_ | 30 (48%) | 32 (51%) | 1 (1%) | 63 |
| **Total P_3_** | **56 (45%)** | **66 (52%)** | **4 (3%)** | **126** |
| Left P_4_ | 10 (16%) | 38 (60%) | 15 (24%) | 63 |
| Right P_4_ | 9 (14%) | 41 (65%) | 13 (21%) | 63 |
| **Total P_4_** | **19 (15%)** | **79 (63%)** | **28 (22%)** | **126** |

Table S5. Results of the antimeric analyses for left and right elements

| Statistical Test | Variable | *p* | α^a^ | *t*-statistic | df^c^ |
| --- | --- | --- | --- | --- | --- |
| P_3_ 3D Data | | | | | |
| Wilcoxon Signed Ranks | Lingual Cusp Number | 0.061 | 0.0500 | 1.877^b^ | 62 |
| Paired t | RICD A-B | 0.747 | 0.0177 | 0.324 | 62 |
|  | RICD A-C | 0.462 | 0.0177 | 0.746 | 27 |
|  | RICD B-C | 0.571 | 0.0177 | 0.573 | 27 |
| P_3_ 2D Data | | | | | |
| Wilcoxon Signed Ranks | Lingual Cusp Number | 0.157 | 0.0500 | 1.414^b^ | 62 |
| Paired t | Crown Area | 0.845 | 0.0125 | 0.196 | 62 |
|  | RICD A-B | 0.870 | 0.0125 | 0.164 | 62 |
|  | RICD A-C | 0.634 | 0.0125 | 0.481 | 27 |
|  | RICD B-C | 0.769 | 0.0125 | 0.297 | 27 |
| P_4_ 3D Data | | | | | |
| Wilcoxon Signed Ranks | Lingual Cusp Number | 0.981 | 0.0500 | 0.024^b^ | 62 |
| Paired t | RICD A-B | 0.516 | 0.0083 | 0.654 | 62 |
|  | RICD A-C | 0.781 | 0.0083 | 0.279 | 48 |
|  | RICD B-C | 0.532 | 0.0083 | 0.629 | 48 |
|  | RICD A-D | 0.687 | 0.0083 | 0.421 | 7 |
|  | RICD B-D | 0.447 | 0.0083 | 0.806 | 7 |
|  | RICD C-D | 0.966 | 0.0083 | 0.044 | 7 |
| P_4_ 2D Data | | | | | |
| Wilcoxon Signed Ranks | Lingual Cusp Number | 0.817 | 0.0500 | 0.232^b^ | 62 |
| Paired t | Crown Area | 0.286 | 0.0071 | 1.076 | 62 |
|  | RICD A-B | 0.321 | 0.0071 | 1.000 | 62 |
|  | RICD A-C | 0.781 | 0.0071 | 0.279 | 48 |
|  | RICD B-C | 0.207 | 0.0071 | 1.279 | 48 |
|  | RICD A-D | 0.992 | 0.0071 | 0.010 | 7 |
|  | RICD B-D | 0.207 | 0.0071 | 1.389 | 7 |
|  | RICD C-D | 0.783 | 0.0071 | 0.286 | 7 |

^a^The overall alpha value is 0.05, recalculated using the Bonferroni correction.

^b^This value is the *z*-score, the Wilcoxon Signed Ranks Test equivalent to the *t*-statistic.

^c^Degrees of freedom.

Table S6. Independent t-test results comparing mean A-B-C cusp angle in two and three lingual cusped teeth (bootstrap resampling applied)

| Tooth | *p*-value^a^ | α-value | *t*-statistic |
| --- | --- | --- | --- |
| P_3_ | **<0.001** | 0.05 | 13.08 |
| P_4_ | **<0.050** | 0.05 | 3.74 |

^a^Statistically significant results (*p*<0.05) are bolded.

# SUPPLEMENTAL FIGURES

#

| Figure S1. Occlusal views of a P4 crown with topographic overlay in MeshLab (**I.**) and ImageJ (**II.**). (**I.**) Cusp tip locations are approximated and marked by black dots with reference to the curvature map and regional maxima along the z-axis with adjustments made for wear or alternative cusp morphology as viewed from the original images. Solid black lines between cusp tips represent intercusp distances. (**II.**) Cusp areas traced using ImageJ’s freehand selection/trace tool, with shading used to distinguish the buccal (blue), mesiolingual (yellow), and distolingual (red) cusps. |
| --- |

| 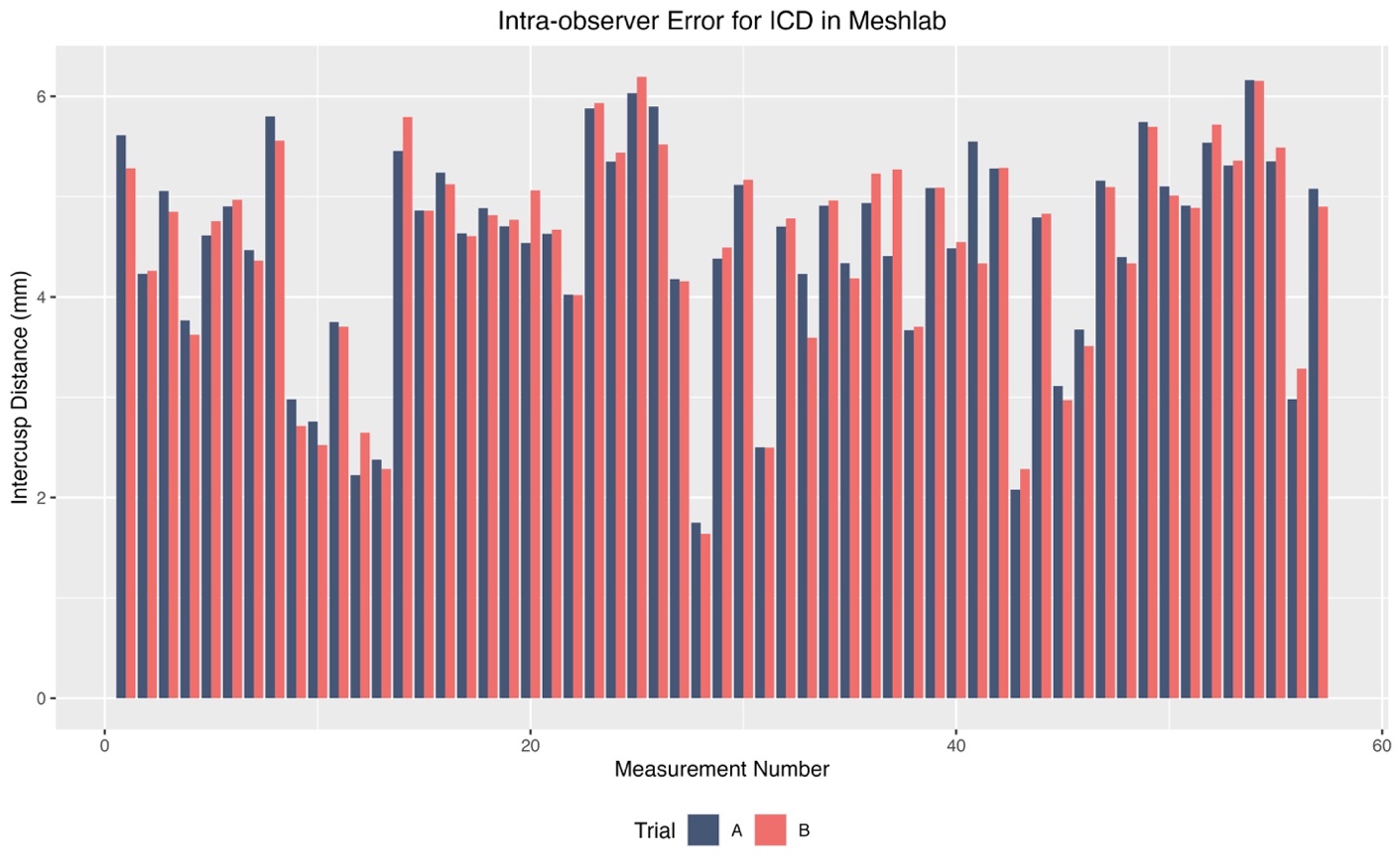 |
| --- |
| Figure S2. Bar graph illustrating raw measurements from the intra-observer error study for ICDs in MeshLab. |

| 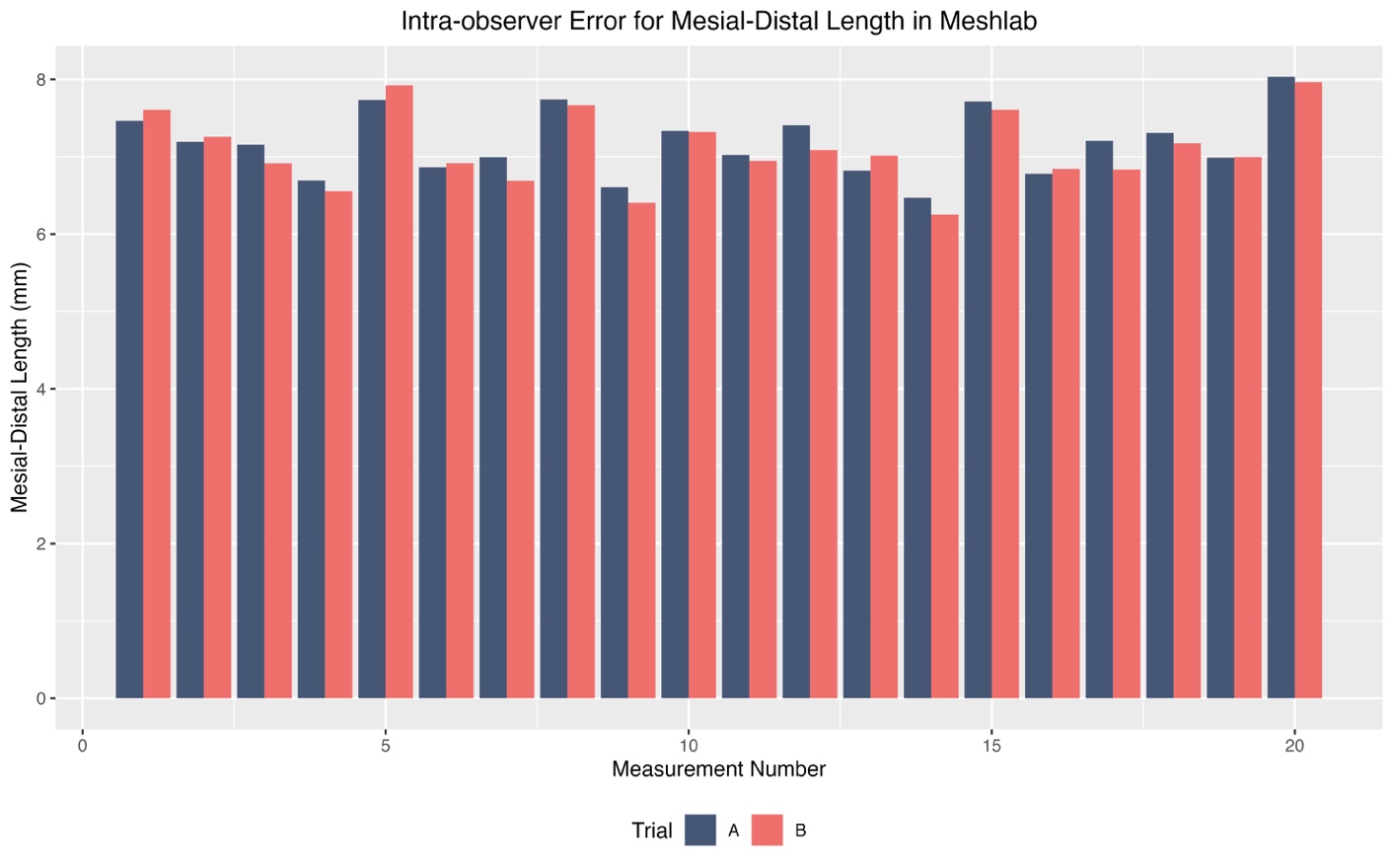  A  B |
| --- |
| 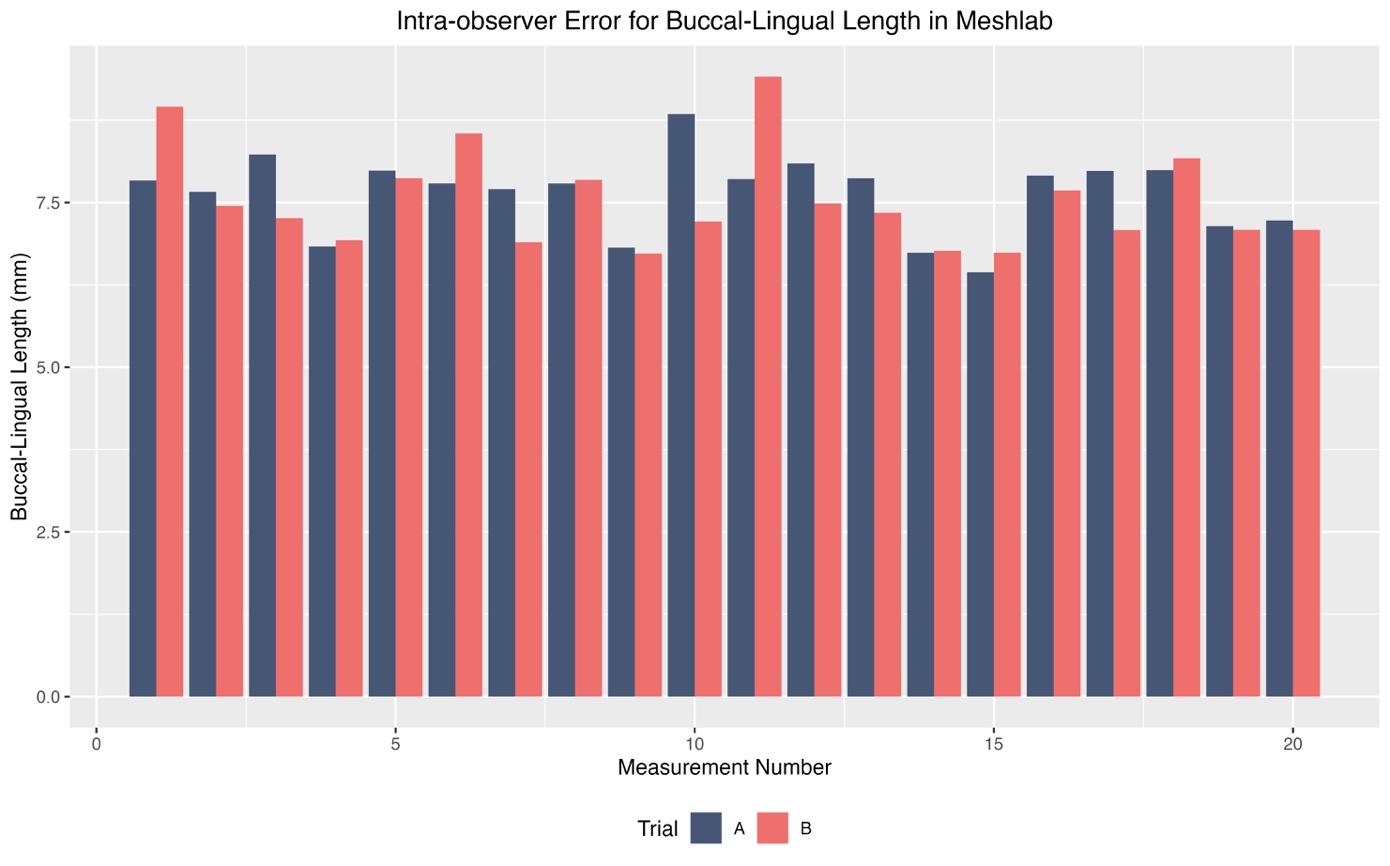 |
| Figure S3. Bar graphs illustrating raw measurements from the intra-observer error study for crown area in MeshLab. Top (**A**) is mesial-distal length, and bottom (**B**) is buccal-lingual length. |

| 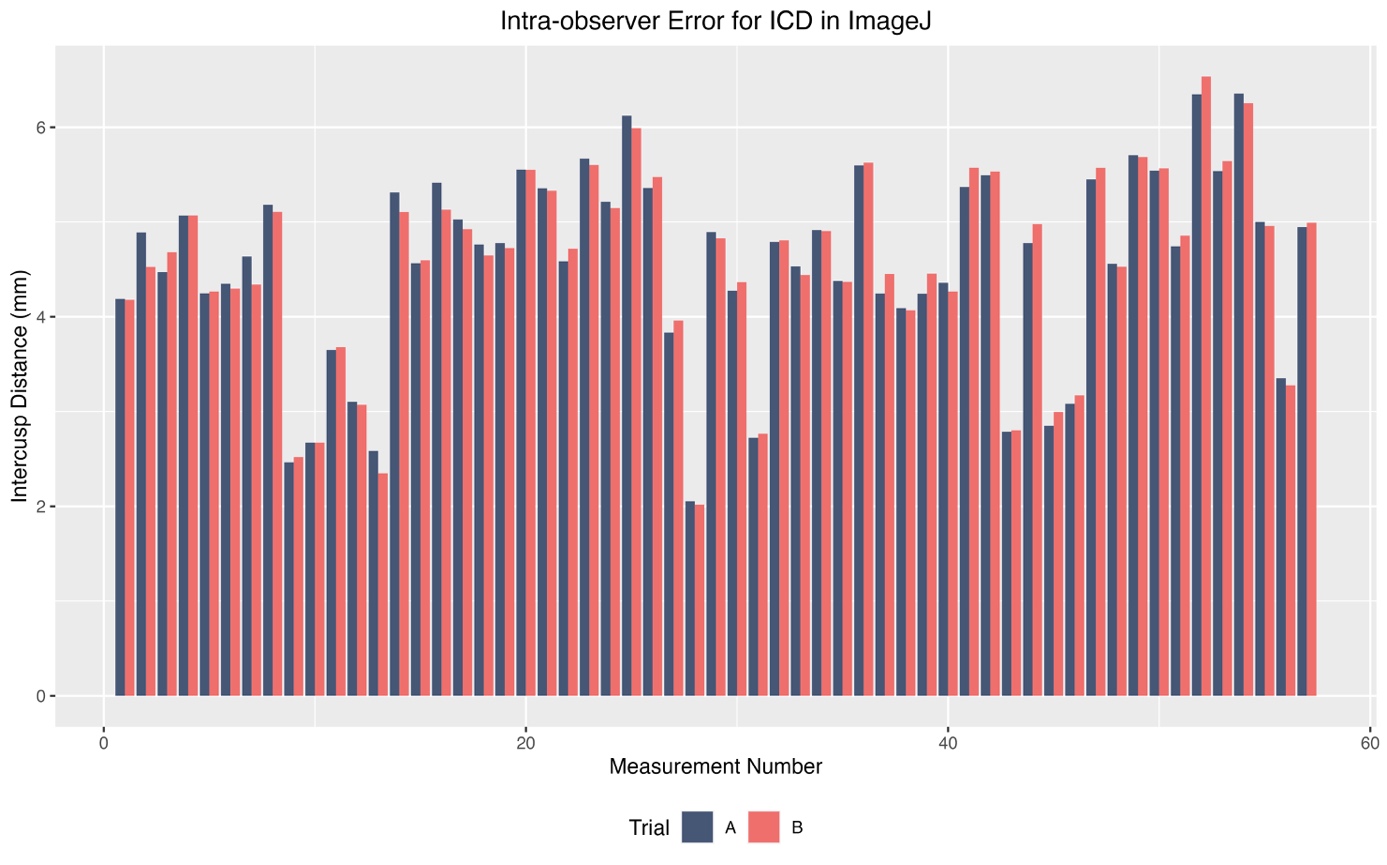 |
| --- |
| Figure S4. Bar graph illustrating raw measurements from the intra-observer error study for ICDs in ImageJ. |

| 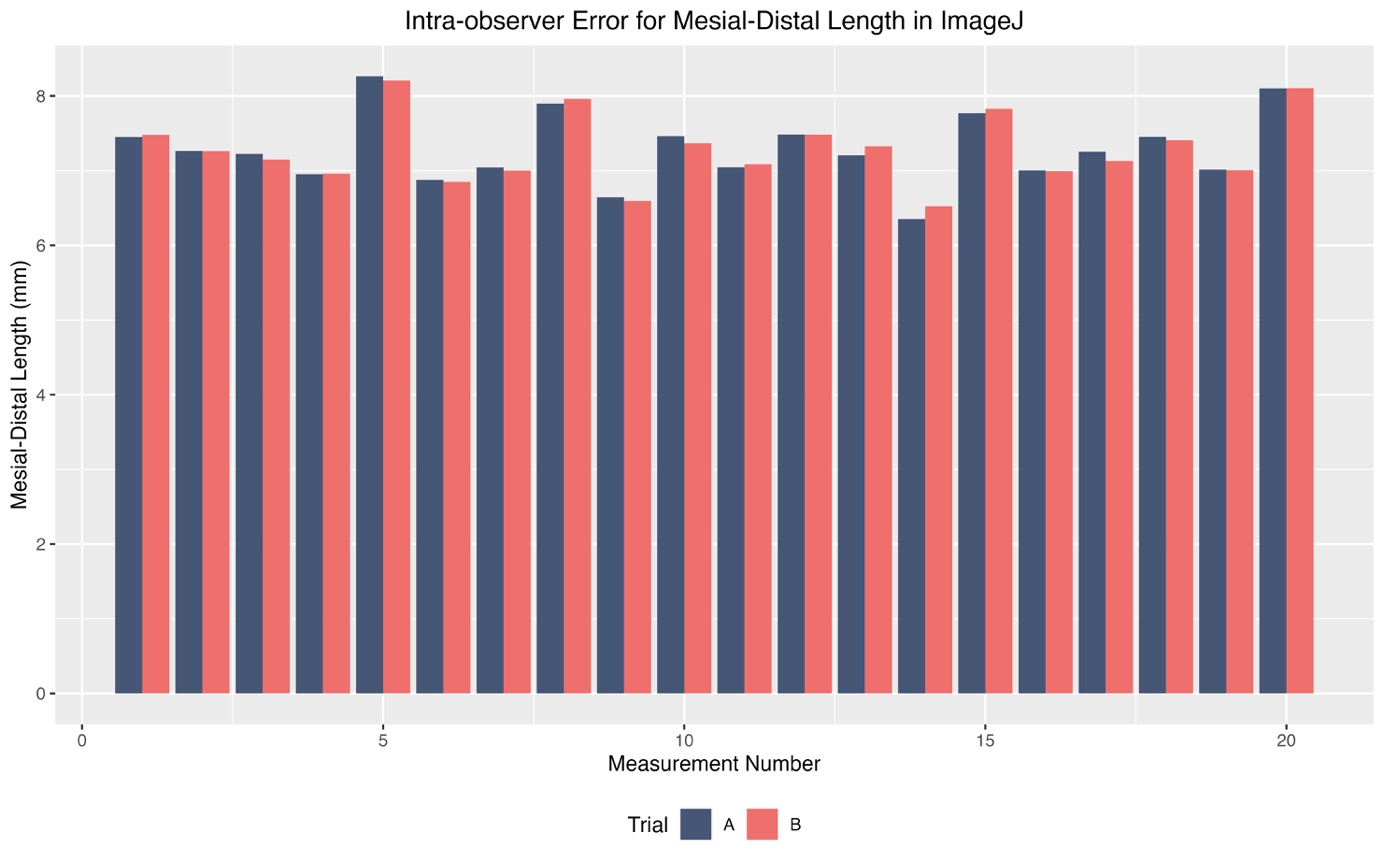  B |
| --- |
| 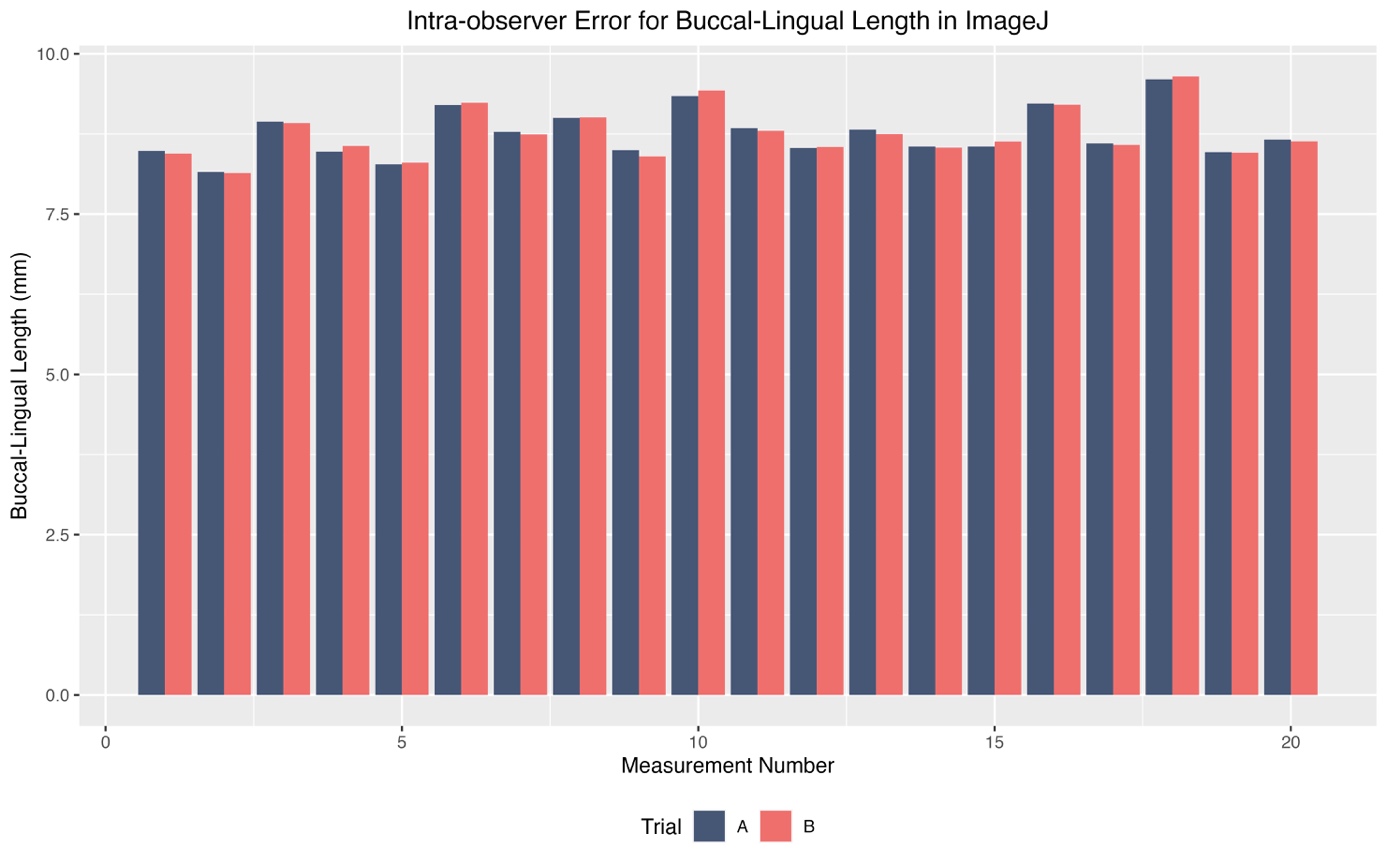 |
| Figure S5. Bar graphs illustrating raw measurements from the intra-observer error study for crown area in ImageJ. Top (**A**) is mesial-distal length, and bottom (**B**) is buccal-lingual length. |

A

| A. |
| --- |
| B. |
| Figure S6. Pie charts comparing (**A.**) P_3_ sample by lingual cusp number, and (**B.**) P_4_ sample by lingual cusp number. |

**
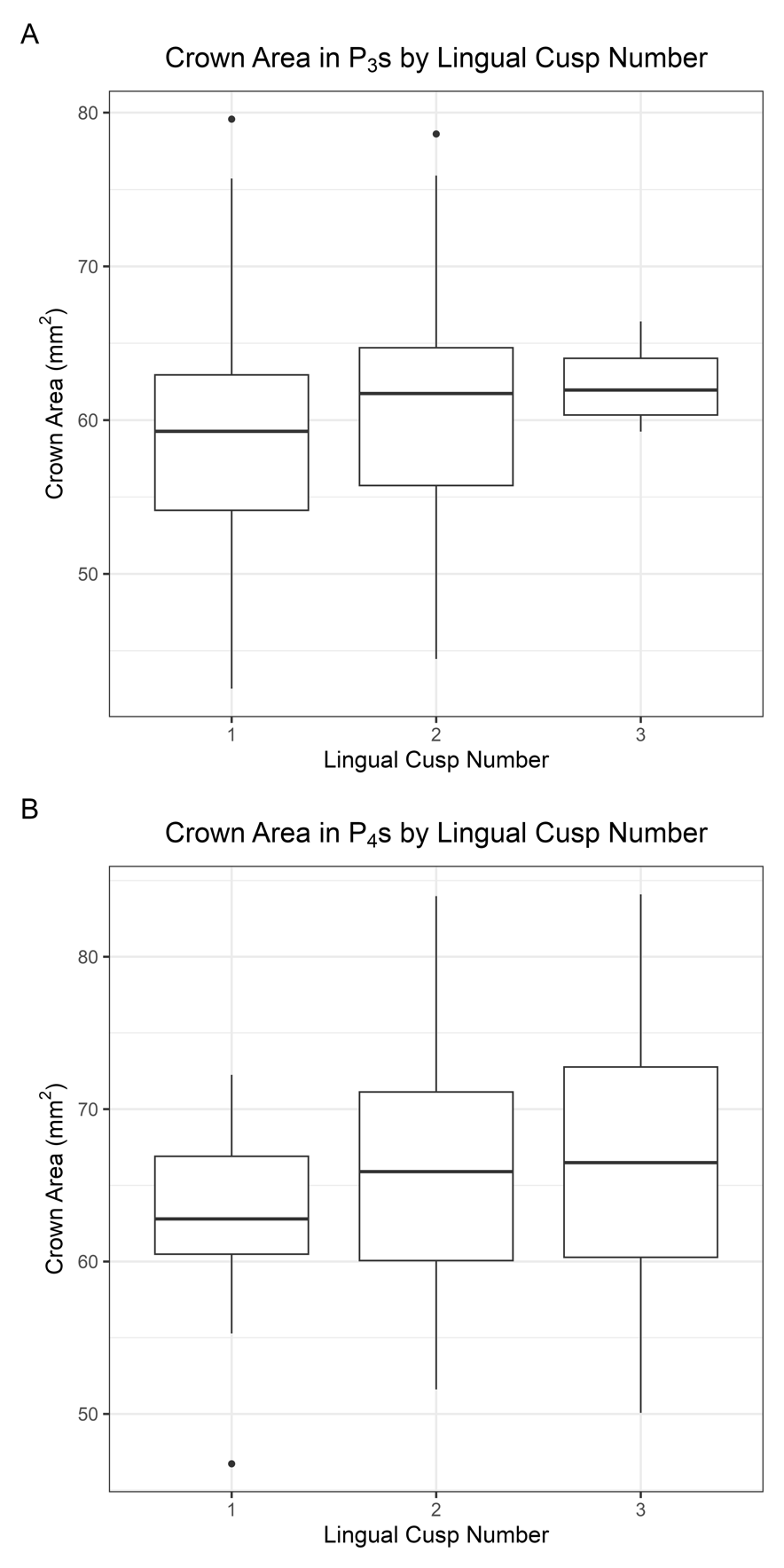
**

Figure S7. Crown area by lingual cusp number for (**A.**) P_3_s, and (**B.**) P_4_s.

| A. |
| --- |
| B. |
| Figure S8. Pie charts comparing (**A.**) two lingual cusped P_4_ by average RCA, and (**B.**) three lingual cusped P_4_ by average RCA. |
